# ^1^H NMR Metabolite Monitoring during the Differentiation of Human Induced Pluripotent Stem Cells Provides New Insights into the Molecular Events That Regulate Embryonic Chondrogenesis

**DOI:** 10.3390/ijms23169266

**Published:** 2022-08-17

**Authors:** Ashley Coope, Zain Ghanameh, Olivia Kingston, Carl M. Sheridan, Richard Barrett-Jolley, Marie M. Phelan, Rachel A. Oldershaw

**Affiliations:** 1 Department of Musculoskeletal and Ageing Science, Institute of Life Course and Medical Sciences, Faculty of Health and Life Sciences, University of Liverpool, William Henry Duncan Building, 6 West Derby Street, Liverpool L7 8TX, UK; 2 Clinical Directorate Professional Services, Aintree University Hospital, Liverpool University Hospitals NHS Foundation Trust, Lower Lane, Liverpool L9 7AL, UK; 3 Department of Eye and Vision Sciences, Institute of Life Course and Medical Sciences, Faculty of Health and Life Sciences, University of Liverpool, William Henry Duncan Building, 6 West Derby Street, Liverpool L7 8TX, UK; 4 Department of Biochemistry, Institute of Systems, Molecular and Integrative Biology, Faculty of Health and Life Sciences, University of Liverpool, Biosciences Building, Crown Street, Liverpool L7 7BE, UK; 5 High Field NMR Facility, Liverpool Shared Research Facilities (LIV-SRF), Faculty of Health and Life Sciences, University of Liverpool, Crown Street, Liverpool L69 7ZB, UK

**Keywords:** ^1^H NMR metabolomics, metabolomics, metabolite, metabolism, induced pluripotent stem cell, human embryogenesis, pluripotency, differentiation, chondrogenesis

## Abstract

The integration of cell metabolism with signalling pathways, transcription factor networks and epigenetic mediators is critical in coordinating molecular and cellular events during embryogenesis. Induced pluripotent stem cells (IPSCs) are an established model for embryogenesis, germ layer specification and cell lineage differentiation, advancing the study of human embryonic development and the translation of innovations in drug discovery, disease modelling and cell-based therapies. The metabolic regulation of IPSC pluripotency is mediated by balancing glycolysis and oxidative phosphorylation, but there is a paucity of data regarding the influence of individual metabolite changes during cell lineage differentiation. We used ^1^H NMR metabolite fingerprinting and footprinting to monitor metabolite levels as IPSCs are directed in a three-stage protocol through primitive streak/mesendoderm, mesoderm and chondrogenic populations. Metabolite changes were associated with central metabolism, with aerobic glycolysis predominant in IPSC, elevated oxidative phosphorylation during differentiation and fatty acid oxidation and ketone body use in chondrogenic cells. Metabolites were also implicated in the epigenetic regulation of pluripotency, cell signalling and biosynthetic pathways. Our results show that ^1^H NMR metabolomics is an effective tool for monitoring metabolite changes during the differentiation of pluripotent cells with implications on optimising media and environmental parameters for the study of embryogenesis and translational applications.

## 1. Introduction

Embryogenesis defines the process during which the single cell zygote develops into the multicellular embryo, through gastrulation and specification of the three germ layers, the patterning of the body plan and the morphogenesis of functional organ systems [1,2,3]. Major cell fate decisions of cell division, apoptosis and commitment to lineage differentiation are highly coordinated through the spatial and temporal activity of conserved growth factor signalling pathways and the regulation of transcription factor networks and epigenetic mediators [1,2,4]. Fundamental to all of these processes is the complex integration of metabolic pathways as energy demand increases to fuel the accelerated cell activities associated with macromolecular biosynthesis, the biogenesis of subcellular organelles and the increase in cell mass required for cell division and the attainment of specialised cell function during differentiation [5,6]. In addition to the potential to produce energy in the form of adenosine-5′-triphosphate (ATP), intermediate metabolites are also substrates within biochemical pathways, themselves forming the building blocks of amino acids, lipids and nucleic acids [5,6,7]. Intermediate metabolites also contribute to the physiological control of REDOX signalling by controlling reactive oxygen species (ROS) activity [6,8], and as substrates for post-translational histone modifications, such as acetylation and methylation, they can influence the activation and suppression of gene activity [9].

Human pluripotent stem cell populations, including human embryonic stem cells (hESC) [10] and human induced pluripotent stem cells (hIPSC) [11], are characterised by the properties of self-renewal and pluripotency, differentiating into all cell lineages of the human body [10,11,12]. Both hESC and hIPSC have been established as model systems that recapitulate the early molecular events of embryogenesis, germ layer specification, lineage commitment and differentiation to functional specialised cell types, thus demonstrating their value in advancing the study of human embryonic development, drug discovery, the modelling of diseases and inherited disorders and the production of cell therapy and tissue-engineered therapeutics [12,13,14,15,16].

Pluripotent stem cells preferentially source energy requirements from aerobic glycolysis, whereby pyruvate is diverted away from entry into the TCA cycle by reduction to the endpoint metabolite lactate [6,17,18,19]. Commonly referred to as the Warburg effect [17,20,21], aerobic glycolysis was first identified as a phenomenon of cancer cell metabolism and is now established as a core metabolic pathway in highly proliferative cell populations, including pluripotent stem cells and pre-implantation embryos [18,22,23,24]. While glycolysis is energetically less efficient than oxidative phosphorylation, it occurs more rapidly when glucose is present in excess and results in an overall greater production of ATP per unit of time [25,26]. 

The preferential metabolism of glucose through aerobic glycolysis is implicated in wider biological roles that couple bioenergetic state to pluripotent cell physiology and phenotype [5,7]. Selective growth advantage is conferred by freeing-up predominant commitment of carbon to TCA cycle-mediated energy production, enabling the creation of biomass by shunting glucose into the pentose phosphate pathway to generate precursor metabolites used within macromolecular biosynthetic pathways [7]. Glutamine metabolism is critical to regulating the pluripotent state through the control of OCT3/4 degradation [27,28] and is vital for maintaining physiologic cellular oxidative phosphorylation by generating metabolic substrates used in energy and biomass metabolism by feeding into the TCA cycle [6,29]. Glutamine is also a precursor metabolite to glutathione, a critical regulator of REDOX activity that suppresses reactive oxygen species (ROS) and the formation of free radicals that damage subcellular organelles and compromise the epigenetic and genomic integrity of the cell [22,27].

Whilst there is a growing body of evidence to underpin the role of metabolic pathway activity in the homeostasis of pluripotent stem cell physiology and regulation of cell fate decisions, little is known about the integration of metabolic pathways during differentiation to a specialised cell lineage and we have sought to address this by investigating changes in metabolite profiles during the directed differentiation of IPSCs to chondrogenic cells [13,15,16,30,31,32]. 

Our three-stage directed differentiation (DD) protocol simulates the developmental pathways that are regulated during embryonic chondrogenesis. DD is performed by temporally supplementing a chemically-defined base medium with varying combinations of growth factors [30]. In Stage 1, wnt3a, graded concentrations of activin-A and then the latter introduction of bone morphogenetic protein (BMP)-4 directs pluripotent cells to a primitive streak/mesendoderm cell population [30,33,34]. Stage 2 drives differentiation from primitive streak/mesendoderm to a mesoderm cell population through continued supplementation with BMP4 and fibroblast growth factor (FGF)-2 and the parallel inhibition of differentiation to endoderm by the removal of wnt3a and activin-A and supplementation with follistatin [30,35,36]. In Stage 3, the differentiation of mesoderm to chondrogenic cells is driven by a graded switch from BMP4 to pro-articular chondrocyte growth and differentiation factor (GDF)-5 [30,37]. At the end of Stage 3 (the completion of DD) three-dimensional cell aggregates composed of rounded chondrocyte-like cells held within a dense extracellular matrix (ECM) are visible throughout the cell cultures with chondrogenic phenotype confirmed by the production of sulphated glycosaminoglycans (sGAG) and cartilage-specific COLLAGEN II [30,38].

^1^H nuclear magnetic resonance (NMR) metabolic fingerprinting and footprinting is evolving to become a sensitive and specific analytical technique to identify and measure emergent and quantifiable changes in small molecules (metabolites) endogenous to the biological system in a high-throughput and statistically robust manner [39,40]. Employing ^1^H NMR metabolomics affords a semi-targeted assay whereby the simultaneous measurement of multiple metabolites within the ^1^H metabolome can be monitored during biological changes [41,42]. The combination of metabolite fingerprinting (intracellular) and footprinting (extracellular/secreted) enables a holistic study of cell function so that we can tease apart responses to highly complex stimuli, such as those that occur during embryogenesis and the differentiation of pluripotent stem cells [43,44,45]. 

We report here the measurement of the changing metabolome as pluripotent hIPSC differentiate through the three stages of primitive streak/mesendoderm, mesoderm and chondrogenic cells. Our results provide insights into the metabolic pathways that are regulated during chondrogenic differentiation and offer a novel technical approach with which to study the molecular events that take place during embryogenesis as well as monitoring the production of specialised and functional pluripotent stem cell-derived cell populations produced for translational applications.

## 2. Results

### 2.1. Confirmation of Directed Differentiation (DD) of IPSCs to Chondrogenic Cells

The morphology of cultures at each stage of DD was characterised by brightfield microscopy (Supplemental Figure S1). Prior to differentiation Day 0, feeder-free pluripotent IPSC cells appeared as a 2D monolayer of tightly associated cells with higher nucleus to cytoplasmic ratio and prominent nucleoli consistent with a pluripotent phenotype. At Stage 1 (day 4) of DD the initiation of loss of pluripotent phenotype and commitment to differentiation was evidenced by weaker cell–cell contact, individual cells were identifiable within the culture, and these had the appearance of being larger and more spread out compared to pluripotent cultures. At Stage 2 (day 11), the phase-bright clusters of cells representative of mesenchymal condensations were visibly distributed throughout cultures, which, by Stage 3 (day 17), had matured further to form 3D cell aggregates. Individual cells within the 3D aggregates had a rounded chondrogenic cell-like morphology and were embedded in lacunae-like structures surrounded by extracellular matrix (ECM). The chondrogenic cell phenotype was confirmed by positive ECM-specific staining of highly-sulphated proteoglycans with safranin O (Supplemental Figure S2) and the positive immunolabelling of cartilage-specific collagen type II (Supplemental Figure S3).

### 2.2. Metabolic Profiles of IPSCs and Stages of Differentiation

Pluripotent and DD stages 1–3 cell extracts exhibited 146 peak bins; 95 bins were annotated to 56 metabolites and the additional 51 unknown bins intracellularly. Spontaneously differentiating (SD) cell extracts were less metabolite-rich and thus fingerprint analysis of SD was limited to only methyl containing (high sensitivity) metabolites (Supplemental Tables S1 and S2).

Extracellular metabolites exhibited 154 peak bins; 133 metabolites were annotated to 46 metabolites and the additional 21 unknown bins extracellularly (Supplemental Tables S1 and S2).

### 2.3. Metabolomics/Phenotypic Comparison of Directed vs. Non-Directed vs. IPSCs

**Figure 1:**
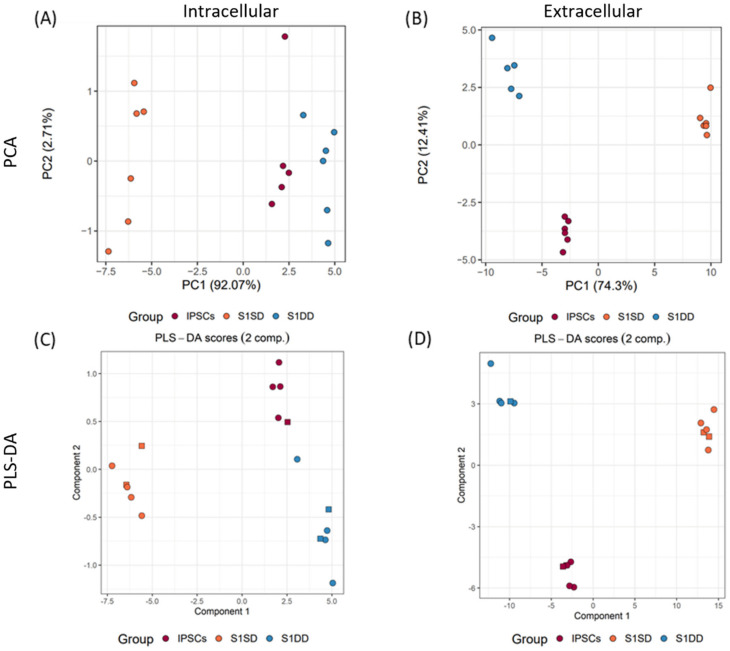
Comparisons of pluripotent and differentiating cell cultures of IPSC, directed differentiation (DD) or embryoid bodies (EB) with DD and EB 4-day post-differentiations by metabolomics. (**A**,**B**) Unsupervised PCA plots of (**A**) intracellular and (**B**) extracellular components. (**C**,**D**) Discriminant two-component PLS-DA plots of (**C**) intracellular and (**D**) extracellular with cross-validated ROCs, each scoring 1. Media sample data adjusted for cell-free controls. Circles or squares indicate the split of data between training and testing, respectively, for predictive PLS-DA models.

The intracellular metabolite profiles of the first stage of differentiation were compared to undifferentiated IPSCs from concurrent cultures. Even at this early stage in the protocol metabolic profiles between stages and differentiation method were clearly distinct on principal component (PC) 1 (explaining 92% of the variance) within the principal component analysis (PCA), with the greatest variance observed between the spontaneously differentiating groups and all other samples (Figure 1A). Extracellular metabolite and nutrient profiles demonstrated similar variance, with unsupervised PCA exhibiting distinct profiles for each group (Figure 1B). 

**Figure 2:**
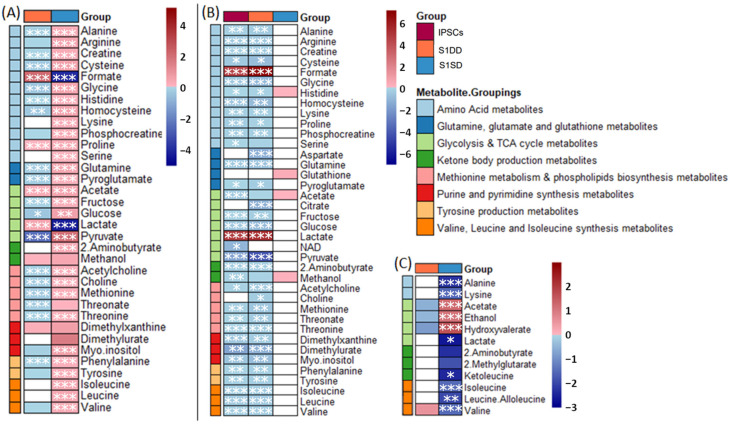
Fold change analysis of key metabolites between pluripotent IPSC and Stage 1 differentiation. (**A**) Intracellular metabolite changes (with baseline corrected to cell-free controls) for DD (blue) or SD (orange) versus IPSC; (**B**) Extracellular metabolite changes directly compared to the respective cell-free controls for IPSC (red) DD (blue) or SD (orange); (**C**) Intracellular methyl-containing metabolites for DD (blue) or SD (orange) versus IPSC samples. For clarity, metabolites are grouped according to key cellular processes. Red cells indicate a fold-change increase, blue cells indicate a fold-change decrease. Cells left white were found to be neither significantly different (<0.05 FDR-adjusted *p*-value) nor with VIP > 1 (by multivariate discriminant analysis). Asterisks indicate FDR-adjusted *p*-values < 0.05 (*), < 0.005 (**), or < 0.001 (***).

To establish the intracellular and extracellular metabolites driving the variance between undifferentiated and Stage 1 differentiated cells, two-component partial least squares discriminant analysis (PLS-DA) models were established (Figure 1C,D). Cross-validation indicated these modest group sized models were able to discriminate (ROC = 1) with variance importance in the projections (VIP) scores of 1 or more, indicative of metabolites influential in these models (Figure 2).

The levels of each of these metabolites were also found to be significantly different by univariate analysis (one-way ANOVA; Supplemental Tables S3–S5). Specifically, the spontaneously differentiating intracellular metabolite profiles when compared to the IPSCs in univariate analyses exhibited significant reductions in lactate that were not observed in the DD cohort (Figure 2C). The spontaneously differentiating cells also demonstrated significantly increased intracellular acetate when compared to the IPSCs, while acetate levels were reduced in the Stage 1 DD samples (Day 4). For the extracellular metabolite profiles acetate was again influential in PLS-DA, with lactate, glutamine, glucose and threonine among other top-ranked VIP metabolites (Figure 2A). 

Metabolite set enrichment analysis (MSEA) on metabolite changes between Stage 1 DD and IPSCs was performed for intracellular and extracellular changes. No pathway was significantly enriched (FDR adjusted *p*-value < 0.05) from the analysis.

Comparing pluripotent IPSCs to either Stage 1 directed or spontaneous differentiation separately highlighted the distinct metabolite profiles for each (Supplemental Table S3). Stage 1 DD exhibited increased extracellular levels of formate, proline and lactate compared to pluripotent IPSCs. Whereas many other amino acids (threonine, phenylalanine, glycine, aspartate, glutamine, histidine, glutamate and cysteine) as well as glutathione, pyruvate and glucose were at comparatively lower levels at Stage 1 than in pluripotent IPSCs. MSEA on extracellular metabolites distinguishing IPSCs from Stage 1 DDs identified several significantly enriched pathways (FDR-adjusted *p*-value < 0.05) (Supplemental Table S4). Extracellular changes between spontaneous differentiation at day 4 compared to pluripotent IPSCs exhibited a much greater distinction. In this analysis the day 4 spontaneous differentiation (equivalent to Stage 1 DD) had comparatively higher levels of almost every metabolite except lactate, which may indicate lower metabolic activity at day 4 spontaneous differentiation. This lower activity was confirmed when metabolite profiles of extracellular media were compared to the cell-free counterpart.

Almost all extracellular metabolites, apart from lactate and formate, were significantly reduced across both the IPSC and DD Stage 1 when compared to the cell-free media indicative of metabolic activity within the cells (Figure 2), i.e., uptake from the media. In contrast, the comparison of extracellular metabolites between spontaneously differentiating cells at day 4 and the cell-free control highlighted very few differences (Figure 2), which reflect the lower metabolic activity in this group.

### 2.4. Metabolomics/Phenotypic Comparison of Spontaneous Differentiation at Timepoints Equivalent to DD Stages 1, 2 and 3

Lower activity of the spontaneously differentiating cells was reflected in the statistical analysis across all timepoints equivalent to each stage of DD with very few metabolite levels significantly changed in comparison to the baseline IPSCs in univariate testing. Extracellular footprints from each timepoint of spontaneous differentiation demonstrated clear differences from each other in unsupervised multivariate analyses (Supplemental Figure S4).

Due to the limited number of intracellular metabolites detected in the spontaneous differentiation, the further monitoring of metabolite changes as differentiation progressed was not possible. Conversely, cell populations undergoing directed differentiation, yielded a higher abundance of metabolites and thus were monitored with metabolic changes analysed up to the completion of Stage 3 (days 17).

### 2.5. Effect of Directed Differentiation at Stages 1, 2 and 3

**Figure 3:**
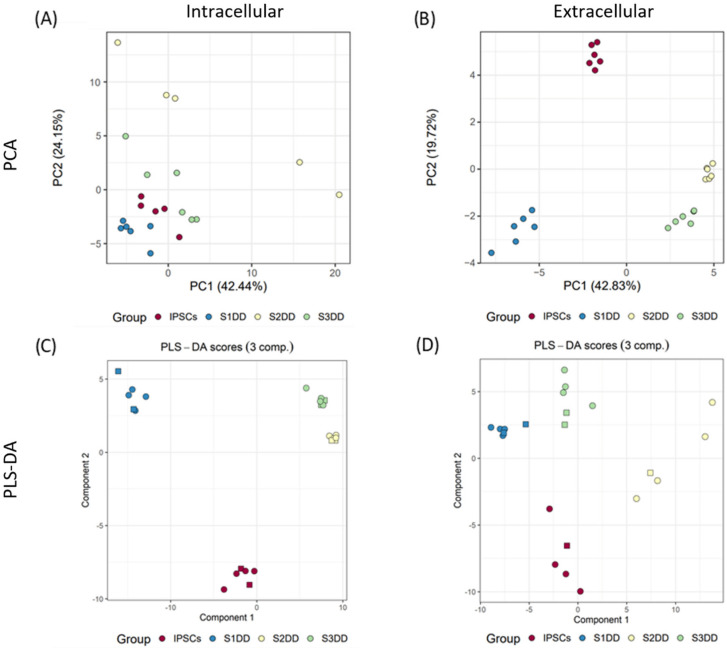
Multivariate analysis of intracellular and extracellular metabolites for pluripotent and directed differentiation Stage 1, 2 and 3. Unsupervised PCAs for intracellular (**A**) and extracellular (**B**) metabolites. Supervised PLS-DA for intracellular (**C**) and extracellular (**D**) metabolites. Circles or squares indicate the split of data between training and testing, respectively, for predictive PLS-DA models.

Comparison of the intracellular metabolite profiles of the DD Stages 1–3 and IPSCs by PCA demonstrated distinct profiles (Figure 3). PCA demonstrated that Stage 2 (day 11) DD samples had higher intragroup variance than the other DD groups, potentially representing an increased heterogeneity of cells at this stage of the protocol. The same distinction between groups was visible when comparing extracellular metabolite profiles albeit with lower intragroup variance. 

As with comparisons between pluripotent IPSC and Stage 1 DD, discriminant models were built to establish the metabolites driving variance between the stages of directed differentiation (Figure 3). Scrutiny of VIP scores and significant variables from one-way ANOVA (Supplemental Table S5) identified the stage-specific fluctuations of intracellular metabolites. 

**Figure 4:**
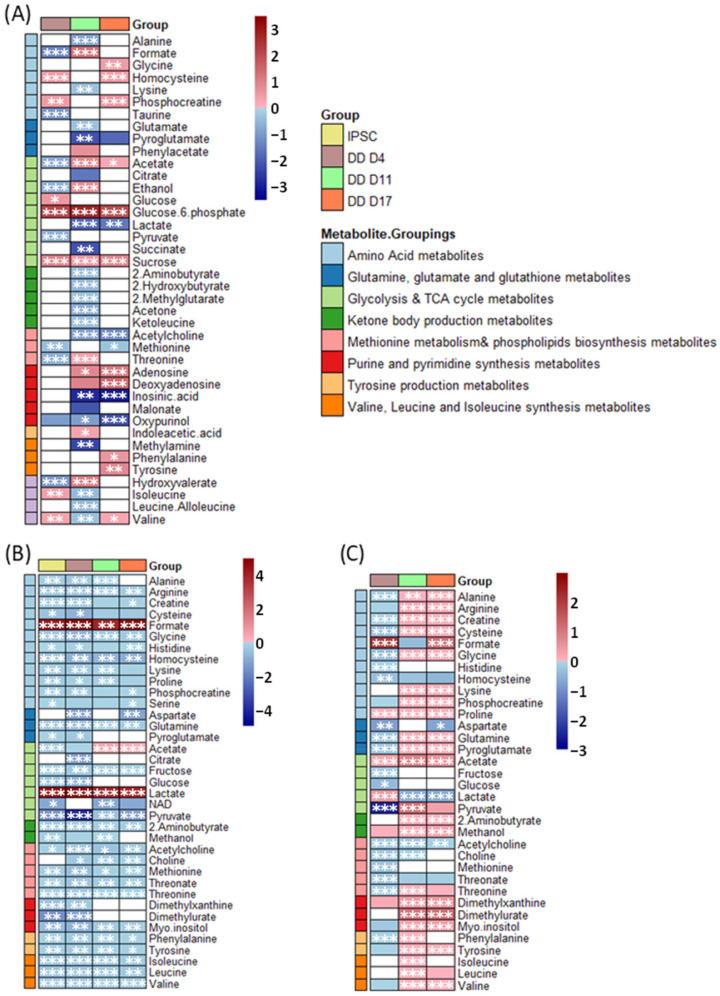
Fold change analysis of key metabolites for pluripotent IPSC and directed differentiation stage 1 (DD D4), 2 (DD D11) and 3 (DD D17). Fold change analysis of (**A**) intracellular metabolites with respect to pluripotent (IPSC) metabolite levels; (**B**) extracellular metabolites with respect to cell-free control; (**C**) extracellular metabolites with respect to IPSC levels. Red cells indicate a fold-change increase, blue cells indicate a fold-change decrease. Cells left white were found to be neither significantly different (<0.05 FDR-adjusted *p*-value) nor with VIP > 1 (by multivariate discriminant analysis). Asterisks indicate FDR adjusted *p*-values < 0.05 (*), < 0.005 (**) or < 0.001 (***).

Many metabolites were comparatively lower at Stage 1 than in pluripotent IPSC but were elevated with respect to IPSC at Stages 2, 3 or 2 and 3, including formate, acetate, ethanol and hydroxyisovalerate (Figure 4A). In contrast, isoleucine was increased at Stage 1 with respect to pluripotent IPSC but then lowered at Stage 2. Many more metabolites either increased with respect to IPSC at later stages (glycine, phenylalanine, tyrosine, phenylacetate, homocysteine, adenosine, deoxyadenosine and indole-acetic acid) or decreased in Stages 2 or 3 with respect to IPSCs (lactate, inosinic acid, malonate, succinate, methylamine, glutamate, pyroglutamate). Sucrose and glucose-6-phosphate were the only two metabolites significantly increased at all stages of DD with respect to IPSCs, and conversely oxypurinol was the only metabolite significantly lower at all stages of DD.

Of all three stages of DD, Stage 2 exhibited the greatest number of intracellular metabolic differences with respect to IPSCs. IPSC metabolite profiles were individually modelled against each stage of DD separately by PLSDA, and VIPs > 1 further demonstrated that metabolites driving discrimination between DD Stages 2 or 3 and IPSC were considerably consistent (acetylcholine, sucrose and glucose-6-phosphate all VIP > 1.4). In contrast, discrimination by PLS-DA between Stage 1 and IPSC extracts were not quite as pronounced for any one metabolite (VIPs of < 1.4) (Supplemental Table S6).

The MSEA of the later stages of DD with IPSCs were also performed. As with the previous comparison of S1 DD with IPSCs, no pathways were found to be significantly enriched (FDR adjusted *p*-value < 0.05).

Extracellular metabolites also varied with DD stage with respect to IPSCs. Glutamine and glucose were influential extracellularly in PLS-DA models (Figure 4C, Supplementary Table S7). Overall, the extracellular metabolites were lower at Stage 1 with respect to IPSC but higher at Stage 2 and 3. Consistently, across all stages of DD with respect to the IPSC extracellular levels of homocysteine, acetylcholine and threonate were observed. In contrast proline, methanol and dimethylxanthine exhibited higher extracellular levels in all DD stages when compared to IPSCs. One-way ANOVA also identified lactate was significantly lower in the Stage 2 and Stage 3 DD samples than in IPSCs. 

Many representative metabolites were significantly reduced across the IPSC and DD stages when compared to the base media (Figure 4B). Among these were notable reductions in glucose, glutamine, methionine, pyruvate and acetate at different stages of differentiation. Formate and lactate were significantly higher in the IPSC and DD bins in comparison to their cell-free controls at all stages. Despite early decreases, acetate was significantly increased in Stages 2 and 3 of the DD protocol compared to the baseline media.

## 3. Discussion

**Figure 5:**
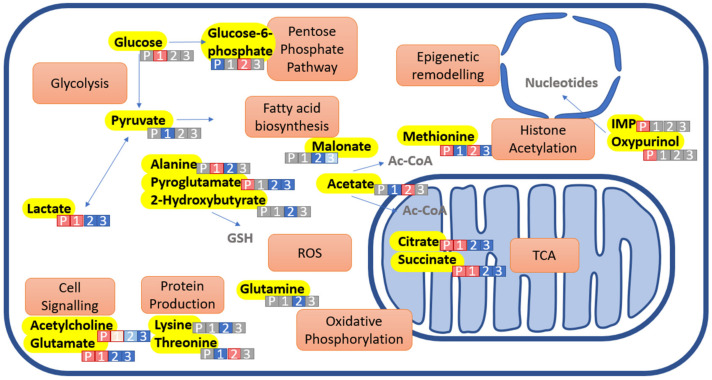
Intracellular metabolite changes between hIPSC, and stages 1, 2 and 3 directed differentiation. Metabolites highlighted yellow are selected metabolite changes observed. Metabolic processes associated are highlighted orange. White lettered/numbered boxes indicate the stage of differentiation (1, 2 or 3) or pluripotent IPSC indicated by ‘P’. Boxes are shaded to indicate the metabolite level at that stage—grey indicates a median value, red indicates an increased level and blue indicates lower level. Molecules in grey text are not observed in this study but are associated. Abbreviations used: GSH, Glutathione; ROS, reactive oxidative species; TCA, tricarboxylic acid cycle; IMP inosine, monophosphate.

We have used ^1^H NMR metabolomics as a novel analytical tool for measuring ^1^H metabolome changes that occur during the directed differentiation of pluripotent IPSC to chondrogenic cells [30]. The key findings of this study were that significant metabolite changes were associated with central metabolism, with aerobic glycolysis being predominant in IPSC, elevated oxidative phosphorylation during differentiation and fatty acid oxidation and ketone body use in chondrogenic cells. We also identified metabolites implicated in epigenetic regulation of pluripotency and differentiation, cell signalling, homeostasis of cell physiology and the activity of biosynthetic pathways (Figure 5).

### 3.1. Pluripotent Stem Cell Metabolism

The IPSC ^1^H NMR metabolic profile was consistent with the established understanding of metabolic flexibility demonstrated by pluripotent stem cells. High levels of pyruvate and lactate were identified in both the intracellular and media components of pluripotent cultures, underpinning their glycolytic phenotype [22,24]. Lactate, conventionally regarded as a waste product of aerobic glycolysis, is now also proposed to be a metabolic substrate through the recycling of carbon into anabolic pathways that generate cellular biomass required for self-renewal [24]. We hypothesise that the future interrogation of lactate flux by ^1^H NMR metabolomics would elucidate a novel mechanism for regulating pluripotency. Carbon shuttling by pentose phosphate pathway may also be indicated by lower levels of glucose-6-phosphate, and this has also been established as a mechanism by which pluripotent stem cells meet the balance of energy production and macromolecular biosynthesis required for the rapid proliferation of an expanding cell population [17]. 

Glutamine was highly abundant in our IPSC cultures, as was expected given its role as a critical metabolite in the maintenance of cellular physiology and the regulation of the pluripotent state [27,28]. Its contribution to cellular oxidative phosphorylation was underpinned by the identification of TCA cycle intermediates (citrate and succinate), which form the pool of metabolic precursors used in biosynthetic pathways and post-translational modifications [6,46]. Glutamine is also required for the generation of glutathione, thereby contributing to the maintenance of REDOX homeostasis [6,8,22,27]. We would expect that ROS and free radical production would be minimised within our IPSC cultures because they were maintained under low oxygen tension, a method shown in previous work to have a positive effect on the health and function of both adult and embryonic stem cells [47,48,49,50,51]. Whilst we identified metabolites involved in glutathione metabolism, namely alanine and pyroglutamate, we did not detect glutathione. Zhang et al. 2017 have previously commented on the role of glutathione in mediating REDOX activity in IPSC, with its overstimulation having a negative effect on cell health by suppressing the activation of other ROS-mediated DNA damage response mechanisms [52]. It may therefore be that our culture method maintained low levels of ROS production such that glutathione was below the limit of detection or else was rapidly turned over in the in the cell before it could reach abundance. 

Acetate was identified within our IPSC cultures, consistent with previous ^1^H NMR analyses of hESC cultures, where it was correlated to levels of acetyl Co-A and a mechanistic role in the regulation of pluripotency through histone acetylation and the maintenance of chromatin structure [18]. The identification of acetate in the medium further correlates with its suggested role as a paracrine mediator that promotes the maintenance of pluripotency across the cell culture [18].

### 3.2. Stage 1 DD (Primitive Streak/Mesendoderm)

Stage 1 of our DD protocol mimics embryonic germ layer specification and represents the early stages of differentiation with loss of pluripotency and transition to a primitive streak/mesendoderm cell population [30]. Our results showed that these early molecular and cellular events are coupled with changes in the ^1^H NMR metabolome, which either persisted through the DD protocol, i.e., were associated with loss of pluripotency (acetylcholine, IMP, lactate, pyroglutamate), or were shown to change again at later stages of DD, suggesting that metabolite turnover was specific to the state of differentiation and cell lineage commitment. 

The evaluation of central metabolism pathways showed a significant increase in metabolic activity coupled with a net uptake of nutrients from the culture medium. The heightened bioenergetic and nutrient demand was attributed to the still highly proliferative cell population broadening its physiological processes to acquire a more motile and migratory phenotype and the commencement of divergent cell functions during germ layer specification and lineage commitment [53,54].

The comparative analysis of metabolites identified between pluripotent IPSC cultures and Stage 1 showed an increase in glucose uptake from the media and a reduction in intracellular pyruvate and the secretion of lactate, which taken together are indicative of an elevation in aerobic glycolysis. The lower levels of intracellular pyruvate at Stage 1 suggest it was more rapidly converted to end-point metabolite lactate as the rate of energy metabolism increased. 

Our analysis also determined a significant decrease in intracellular acetate between pluripotent IPSC and Stage 1 DD and these results were consistent with previous work that reported the reduction of intracellular acetate with early exit of pluripotency, underpinning its role as a critical regulator of the balance between self-renewal and differentiation [18]. Decreased intracellular acetate and histone acetylation has been shown to be concomitant with the down regulation in OCT3/4 RNA and protein expression that can be blocked in a dose-dependent manner by the exogenous supplementation of acetate [18]. The metabolic regulation of epigenetic state through post-translational chromatin modification and the cadence of transcription factor access to DNA binding sites is critical for moderating the self-renewal and differentiation of pluripotent cells [18,55,56]. Further scrutiny of the ^1^H NMR metabolomic dataset identified metabolites that might be associated with DNA histone methylation and S-adenosylmethionine (SAM) cycling, including methionine, threonine and formate, but these were not significantly changed between pluripotent IPSC and Stage 1 of DD to allow us to predict with any confidence a role for histone methylation in the regulation of DD in our protocol. 

### 3.3. Stage 2 DD (Mesoderm)

Stage 2 of our directed differentiation protocol drives the differentiation of cells from primitive streak/mesendoderm to mesoderm [30]. The variance within metabolite levels for sample replicates was increased at this stage, which may be expected with the level of cellular remodelling taking place. As such, the number of significant changes at this stage may be reduced, although some were still observed. 

Metabolite changes between Stage 1 (primitive streak/mesendoderm) and Stage 2 (mesoderm) reflected a further shift in cellular bioenergetics. Pyruvate was significantly increased between Stage 1 and Stage 2, consistent with it being a key metabolic promoter of differentiation to mesodermal cell lineages [57,58]. The increase in pyruvate also correlates with previous studies that report the maturation of mitochondria in differentiating hESC as being at a stage equivalent to mesoderm formation [59]. Increased pyruvate and acetate (the precursor of acetyl Co-A) within our Stage 2 cultures may therefore reflect the bioenergetic shift in favour of oxidative phosphorylation, and this is further supported by the increase in levels of taurine required to maintain the health and function of mitochondria [60] and the significant decrease in lactate corresponding to a reduction in aerobic glycolysis. In contrast, the intermediate metabolites of the TCA cycle (citrate and succinate) and glutamine and pyroglutamate were lower at Stage 2 compared to Stage 1 of DD, which might indicate a greater turnover of these metabolites with the accelerated rate of oxidative phosphorylation-mediated ATP production, though metabolites associated with electron transport chain and REDOX activity were below the limit of detection.

### 3.4. Stage 3 (Chondrogenic Cells)

Stage 3 of our DD protocol promotes differentiation to a chondrogenic cell type of which ~98% of the population are positive for the transcription factor SOX9 [30], and this homogeneity was reflected by the much-reduced intra-group variance in detected metabolite levels when compared to Stage 2. 

Stage 3 was marked by a significant reduction in glucose and acetate, indicative of a reduction in glucose metabolism in the cells. The observed change in intracellular glucose levels was not expected as the base culture medium for differentiation contained supra-physiologic concentrations of glucose (17.5 mM) and was changed every 24 h. While articular chondrocytes rely primarily on glycolysis, oxidative phosphorylation is required to maintain chondrocyte homeostasis with the elevated levels of succinate having been shown to stabilise hypoxia-inducible factor-1α (HIF1α), a transcription factor that directly binds to the SOX9 promotor to regulate the chondrogenic phenotype [61,62]. Furthermore, the impairment of the TCA cycle is implicated in disease phenotypes, such as osteoarthritis [63,64,65]. Significantly, we detected increased the levels of acetone and 2-hydroxybutyrate between Stage 2 and Stage 3 of DD and suggest that these may provide an alternate energy source via the fatty acid oxidation and formation of ketone bodies that feed into the TCA cycle [66]. Previous work in chondroprogenitor mesenchymal stem cells has described the preferential use of ketone bodies over glucose metabolism as a mechanism of reducing deleterious ROS production, and we propose that this catabolic mechanism was active in our Stage 3 DD cultures, which was carried out at the same oxygen tension as the Board et al. 2017 study [67]. 

Significant increases in amino acids were also reported, including valine, tyrosine, alanine, pyroglutamate and lysine, with these returning from levels that had previously been reduced at Stage 2. Metabolite changes detected at Stage 3 implied elevated biosynthetic pathway activity, consistent with the highly anabolic phenotype of chondrogenic cells that are responsible for the synthesis and assembly of the large macromolecular extracellular matrix components that confer the biomechanical properties of the articular cartilage tissue [64,68,69]. 

### 3.5. Strengths, Weaknesses and Limitations

Our study has demonstrated the application of ^1^H NMR metabolomics as a tool for profiling the changing metabolome during the directed differentiation of IPSC to a chondrogenic cell population. Metabolite profiles identified within this study would be dependent on the chemical composition of the media used, including glucose and glutamine concentration, and also the oxygen tension under which the cells were cultured. The interpretation of the results is within the context of experimental design, although extrapolation of our findings will facilitate the design of optimal media and culture systems that promote the health and differentiation of pluripotent cells and the development of strategies that translate innovations, such as the study of embryo development and in vitro fertilisation technologies, drug screening assays, disease modelling and cellular therapies. 

As extracellular composition changes between stages, great care was taken to control with identical medium cultured in the absence of cells under identical conditions. Medium composition at each stage of directed differentiation is identical in small molecule composition (albeit with different growth factors, Supplemental Table S8). These growth factors exhibited no direct changes on the extracellular metabolite profiles; however, IPSC medium is proprietary and as such does contain different nutritional make-up. As such we have limited our interpretation of extracellular changes to accommodate for the necessary differences in medium composition at each stage of differentiation.

The robust comparative analysis of spontaneous differentiation was prohibited in this study by the limits of detection within EB cultures. Metabolites that were identified showed few differences between intracellular and extracellular components, indicating little nutrient uptake from the culture medium and low metabolic activity within the cells. The further optimisation and scaling of cultures is required for the evaluation of spontaneous differentiation and single cell chemometric and genetic analysis would provide a biological context to a cell culture that would be more diverse and heterogeneous than that generated during directed differentiation strategies. 

## 4. Materials and Methods

### 4.1. Ethical Approvals

UKKi026a (derived from a peripheral blood mononuclear cell) was obtained from the European Bank for Induced Pluripotent Stem Cells (EBiSC). A Material Transfer Agreement (MTA) for the use of this cell line in academic research is in place with the UK Health Protection Agency. 

### 4.2. Cell Culture

#### 4.2.1. Maintenance of IPSCs

Detailed protocols describing the recovery, maintenance and cryopreservation of the UKKi26a IPSC line are described in ‘EBiSC Protocol for the Use of Induced Pluripotent Stem Cells’ (EBiSCProtocolforuseofiPSCv3.pdf; accessed on 10 May 2021). UKKi026a was maintained by culture on vitronectin substrate (0.5 µg/cm^2^, Thermo Fisher Scientific, Warrington, UK) in Stemflex™ culture medium (Gibco™, Thermo Fisher Scientific) in a humidified cell culture incubator at 37 00B0C, 5% CO_2_, 5% O_2_. At confluence, cells were passaged at a ratio of 1:3 using 0.5 mM EDTA. 

#### 4.2.2. Directed Differentiation of (DD) IPSCs to Chondrogenic Cells

IPSCs were sub-cultured onto 6-well culture dishes coated with 50 µg/mL fibronectin (R&D Systems, Abington, UK) in Stemflex™ culture medium and cultured as a monolayer to 90% confluence. The protocol for DD is described in Supplemental Table S8. Cells were cultured on defined matrix substrates in a basal medium (DMEM:F12, 2 mM L-glutamine, 1% (*v*/*v*) ITS, 1% (*v*/*v*) nonessential amino acids, 2% (*v*/*v*) B27, 90 μM β-mercaptoethanol; all reagents from Gibco™, Thermo Fisher Scientific) temporally supplemented with growth factor combinations. Medium changes were performed every 24 h as outlined. Gelatin was purchased from Sigma, Poole, UK. All growth factors were purchased from R&D Systems. 

Differentiating cultures were harvested for ^1^H NMR metabolomic analysis at day 0 (pluripotent cells), day 4 (Stage 1, equivalent to mesendoderm), day 11 (Stage 2, mesoderm) and day 17 (Stage 3, chondrogenic cells). Confirmation of chondrogenic phenotype was carried out at day 17.

#### 4.2.3. Spontaneous Differentiation of IPSCs via Embryoid Bodies

Embryoid bodies were formed by detaching IPSCs from vitronectin-coated tissue culture plastic using 0.5 mM EDTA and culturing as a suspension culture (bacteriological dishes) in EB medium (DMEM, 10% *v*/*v* FBS). 

#### 4.2.4. NMR Metabolomic Controls

Cell-free controls for each cell population were established in parallel with matrix and media changes performed exactly in line with plates containing cell culture. This established that any background molecular signature arising from plastics, matrix and media is constant across all experiments (Supplemental Figure S5).

### 4.3. Immunofluorescence and Histological Analyses

#### 4.3.1. Immunofluorescence for Collagen II

Cell cultures were fixed with paraformaldehyde (PFA; 4% (*w*/*v*) PFA (Sigma) in PBS, pH 7.4, (Gibco™, Thermo Fisher Scientific)) at 4 °C for 20 min. Fixed cell cultures were incubated in blocking buffer (0.1% (*w*/*v*) bovine serum albumin (BSA; Sigma), 10% (*v*/*v*) goat serum (Sigma) in PBS) for 1 h at room temperature before overnight incubation at 4 °C with primary mouse anti-human collagen II antibody (anti-COL2A1 (M2139): sc-52658, Santa Cruz Biotechnology, Heidelberg, Germany) diluted to 10 µg/mL in blocking buffer. Cell cultures were rinsed 3 times with PBS and then incubated for 1 h at room temperature with goat anti-mouse IgG (H + L) cross-adsorbed secondary antibody, Alexa Fluor^®^ 488 (A-11001, Thermo Fisher Scientific) diluted from commercially supplied stock to a ratio of 1:500 in blocking buffer. Control cultures were treated in parallel as a primary isotype control (mouse IgG isotype control (10 µg/mL), Thermo Fisher Scientific) and no secondary antibody control. Cell cultures were counter-stained with 300 nM 4′,6-diamidino-2-phenylindole (DAPI) for 5 min (Sigma). Images were acquired by Zeiss Axio Observer Z1 inverted fluorescent microscope (DAPI, excitation 353/emission 465; Green, excitation 493/emission 517). 

#### 4.3.2. Safranin O Staining for Sulphated Glycosaminoglycan (sGAG)

Cell cultures were rinsed 3 times with PBS before incubation with safranin O stain (0.1% (*w*/*v*) safranin O (in 0.1% (*v*/*v*) in acetic acid) for 20 min at room temperature. Cell cultures were washed extensively with PBS until the rinse was clear.

### 4.4. NMR Metabolomic Analysis

#### 4.4.1. Harvesting of Cell Cultures for NMR Metabolomic Analysis

Media from cell cultures and cell-free controls was transferred in 1 mL aliquots to 1.5 mL microfuge tubes and centrifuged at 700× *g* for 3 min. Supernatants were transferred to fresh, snap frozen in liquid nitrogen and stored at −80 °C,

Adherent cell cultures from DD were rinsed 3 times in Dulbecco’s phosphate buffered saline with calcium and magnesium (DPBS, Ca^++^/Mg^++^; Thermo Fisher Scientific). After the removal of the third wash, 1mL aliquots of (DPBS, Ca^++^/Mg^++^) were transferred to the culture dish and the adherent cells scraped into solution using a pipette tip. Cells were transferred 1.5 mL microfuge tubes and collected by centrifugation at 700× *g* for 3 min. DPBS, Ca^++^/Mg^++^ supernatant was removed before snap freezing the cell pellets in liquid nitrogen and storing at −80 °C.

EBs were transferred from suspension cultures in to 1.5 mL microfuge tubes and collected by centrifugation at 700× *g* for 3 min. EBs were rinsed 4 times in (DPBS, Ca^++^/Mg^++^). After removal of the fourth wash, EBs were collected by centrifugation and the DPBS, Ca^++^/Mg^++^ supernatant removed before snap freezing the EB pellets in liquid nitrogen and storing at −80 °C.

#### 4.4.2. Extraction of Intracellular Metabolites

Cell pellets were retrieved from −80 °C directly onto dry ice. Metabolites were preserved by thawing the cell pellets on wet ice into 500 µL volumes of ice-cold NMR intracellular metabolite buffer (50% (*v*/*v*) HPLC-grade acetonitrile (Fisher Scientific, Loughborough, UK) in double-distilled water (ddH_2_O)). Samples were homogenised by sonication over an ice bath at 3 × 30 s pulses with a 30 s pause between pulses. Samples were vortexed for 15 s and centrifuged at 21,500× *g* for 5 min at 4 °C. The supernatant was collected in a fresh 1.5 mL microfuge tube, snap frozen in liquid nitrogen and lyophilised overnight (at −55 °C, Thermo Scientific, Cheshire, UK). Lyophilised samples were stored at −80 °C [70]. 

#### 4.4.3. Preparation of Samples for NMR Analysis

All reagents were purchased from Sigma unless otherwise stated. 

Media samples were retrieved from −80 °C and thawed at room temperature. Media volumes of 330 µL were transferred to 1.5 mL microfuge tubes with the addition of NMR extracellular acquisition buffer (1 M sodium phosphate buffer, 1.2 mM sodium azide in 20% deuterated water (2H_2_O; all chemicals from Sigma)). Samples were vortexed for 30 s, centrifuged at 21,500× *g* for 5 min at 4 °C and then transferred to a 5 mm NMR tube (Bruker Labscape, Coventry, UK) for NMR acquisition [70]. 

Lyophilised intracellular metabolite samples were retrieved from −80 °C and reconstituted in 200 µL volumes of NMR intracellular acquisition buffer (100 mM sodium phosphate, 0.1% (*v*/*v*) 3-(trimethylsilyl-2,2′,3,3′-tetradeuteropropionic acid (TSP) in 2H_2_O). Samples were vortexed for 30 s, centrifuged at 21,500× *g* for 5 min at 4 °C and then transferred to a 5 mm NMR tube for NMR acquisition [70].

#### 4.4.4. NMR Set-Up and Acquisition

^1^H NMR acquisitions were completed on a 700 MHz Avance III HD spectrometer equipped with a TCI cryoprobe and a chilled ‘SampleJet’ Autosampler (Bruker, Coventry, UK). Carr–Purcell–Meiboom–Gill (CPMG) presaturated standard vendor-supplied pulse sequence (cpmgpr1d) was employed for the attenuation of any large molecular weight components for optimal quantification. All experimental details and spectral parameters are available at the MetaboLights database (www.ebi.ac.uk/metabolights/MTBLS4854; accessed 15 August 2022) [71]. Briefly, spectra were acquired with a 4 s interscan delay, 32 (extracellular) or 256 (intracellular) transients at 25 °C. Spectra conditions were optimised with standard 3-Dimensional shimming and temperature calibration within 0.1 °C [72].

#### 4.4.5. Spectral Processing and Quality Control

The spectra were preprocessed at the spectrometer to apply a Fourier transformation, correcting for deviations in phasing and baseline using standard vendor-supplied routines (apk0.noe). Intracellular and extracellular spectra acquired in this study are presented in Supplemental Figure S6. Tissue extract spectra were aligned directly to TSP at 0 ppm, while spectra from media samples were aligned indirectly via anomeric glucose peak at 5.24 ppm. The quality control (QC) of the spectra was manually performed in accordance with Metabolites Standard Initiative (MSI) recommendations [73]. These criteria included review of the baseline deviation, line width, signal-to-noise ratio and the water suppression of spectra. Where possible, samples which failed QC were replaced with results from replicate samples.

#### 4.4.6. Metabolite Annotation

The annotation of spectral peaks was achieved using a combination of in-house metabolite pattern files and Chenomx standard spectra (Chenomx Inc., Edmonton, AB, Canada). Custom pattern files for the cell and media extracts were generated by adjusting individual ppm peak boundaries for each annotation. Resultant pattern files were uploaded alongside their representative spectra and subject to spectral bucketing using tameNMR. Cell extract spectra were integrated into 165 bins, of which 95 (57.57%) of annotations represented the 56 known unique metabolites. For EBs where signal to noise was less favourable the methyl-containing metabolites were annotated leading to a reduced number of representative metabolites (n = 12) and bins (n = 28). Media spectra yielded 167 total bins, with 138 (82.63%) of the annotations including 41 unique metabolites. For cell and media spectra, 46 and 31 bins were annotated as including unknown metabolites, respectively. Binned spectra, their metabolite annotations and associated pattern files are available via MetaboLights (MTBLS4854) [71]. Where multiple bins were annotated with a single metabolite, an in-house correlation scoring metric (CRS) script was applied to select the most representative bin for each metabolite for use in further analyses [74]. Where correlation scores were low between bins or scores were tied, bins were manually examined to determine the most representative bin. Factors in the selection process included signal-to-noise ratio, presence of overlapping spectra and the Chenomx visualisation of relevant bins.

#### 4.4.7. Statistical Analysis

Binned spectral tables integrated from the cell and media datasets acquired in this study were imported into R (Version 4.1.2, R Core, Team, the R foundation for statistical computing, 2021, Vienna, Austria, r-project.org) for univariate and multivariate analyses using in-house scripts. Raw count data for each dataset were first visualised in R plots to facilitate further QC tests. Bins with significant skew across all samples due to water suppression were eliminated, with n = 146 and n = 154 bins taken forward to normalisation in the cell and media datasets, respectively. Raw counts for cell data in the EB groups demonstrated poor signal-to-noise when compared to other cell samples. As such, only bins representative of methyl spectra from EB cell samples (1.9828–1.9021 ppm and 1.569–0.7279 ppm) were taken forwards to normalisation. Raw count data was visualised using Metaboanalyst (version 5.0, metaboanalyst.ca, Xia Lab, Edmonton, AB, Canada) and R generated Q-Q plots to select appropriate means of normalisation [75]. Each dataset was then normalised by Total Area [70]. To assess the effect of normalisation on each sample, scaling factors were calculated for the cell dataset with all bins, and the methyl bins only. Scaling factors were maintained across cell samples, regardless of whether the whole dataset or methyl data was used, permitting analyses including only methyl bins (Supplemental Table S9). Where the normalisation of data created negative values due to low count scores, affected bins were imputed to values of 100, facilitating Log2 transformation for multivariate and fold-change analyses.

Univariate analyses were performed separately for the normalised datasets of media samples, cell samples excluding EB data and methyl bins for all cell samples. The analyses compared stages of the differentiation protocol using one-way ANOVA testing and Benjamini–Hochberg (FDR) correction for multiple (Padj < 0.05 for statistical significance). Post hoc Tukey values were used to determine significant comparisons with results filtered for metabolite representative bins. Log2 transformed counts for each dataset were mean-centred and Pareto scaled for multivariate analyses [76]. Principal component analyses (PCA) facilitated the identification of potential outliers, while partial least squares discriminant analysis (PLS-DA) models were used to identify metabolites accounting for variance between differentiation stages. PLS-DA models were trained with a random 70% sample of cell extract or media metabolite profiles and were subsequently tested with the remaining 30% of cell extract or media metabolite profiles. The split of data between training and testing are indicated by circles or squares, respectively. PLS-DA models were validated using receiver operator characteristic (ROC) scores [77] and 2000 permutations per model (Supplemental Table S7). Ten-fold cross validation (CV) was performed per PLS-DA to calculate accuracy; R2, Q2 and train:test splits were employed to determine AUROC as per Szymanska et al. 2012 [78]; and 2000 permutations of each PLS-DA were performed via metaboanalyst (Xia et al. 2009 [79]). Summary statistics for all PLS-DA models are presented in Supplemental Table S10. Metabolite representative bins with a Variable Importance in Projection (VIP) score above 1.00 were considered VIP metabolite representative scorers.

#### 4.4.8. Metabolite Set Enrichment Analysis (MSEA)

Lists of VIP metabolite representative scorers with statistical significance in each of the univariate comparisons were input to Metaboanalyst (version 5.0, metaboanalyst.ca, Xia Lab, Edmonton, AB, Canada) and analysed for overrepresentation using the enrichment feature [74]. Results for SMPDB and KEGG pathways were examined to determine and compare metabolic processes for each group. Boxplots were produced in R to examine key metabolites within enriched groups.

## 5. Conclusions

We validated 1H NMR metabolomics as a tool for monitoring intracellular and extracellular metabolite changes throughout directed and spontaneous differentiation from Pluripotent cells. In addition to the specific intracellular metabolite changes observed with loss of pluripotency, the increased variance during differentiation highlights the heterogeneity of differentiating cell populations, specifically at Stage 2 of chondrogenic differentiation.

## Data Availability

All experimental details, spectral parameters, binned spectra, their metabolite annotations and associated pattern files are available at the MetaboLights database (www.ebi.ac.uk/metabolights/MTBLS4854) [71].

## Supplementary Materials

The supporting information can be downloaded at: https://www.mdpi.com/article/10.3390/ijms23169266/s1.
