# Supplementary material for: 1H NMR Metabolite Monitoring during the Differentiation of Human Induced Pluripotent Stem Cells Provides New Insights into the Molecular Events That Regulate Embryonic Chondrogenesis
Source: Int J Mol Sci. 2022 Aug 17;23(16):9266. doi: 10.3390/ijms23169266 (PMC9409419; doi:10.3390/ijms23169266)
Supplement: Supplementary file 1 [file ijms-23-09266-s001.zip › ijms-1799647-supplementary.pdf]

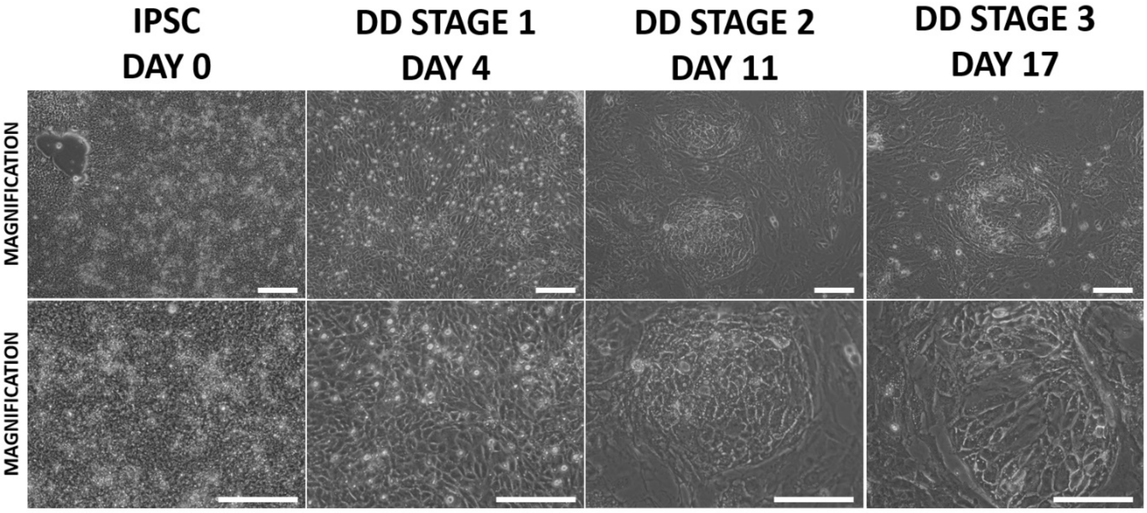

Supplemental Figure S1. Morphology of cultures during directed differentiation (DD) of UKKi26a to chondrogenic cells. Pluripotent UKKi26a cells maintained as feeder-free cultures formed a 2D monolayer of tightly-associated cells with high nucleus to cytoplasmic ratio and prominent nucleoli. At Stage 1 / day 4 of DD differentiating cells appeared more loosely associated, were larger and more spread-out. At Stage 2/ day 11 of DD mesenchymal condensations of cells were distributed through the cultures, which at Stage 3 / day 17 had matured to form 3D aggregates. Individual cells within the 3D aggregates had a rounded chondrogenic cell-like morphology and were embedded in lacunae-like structures surrounded by extracellular matrix. Scale bars = 50μm.

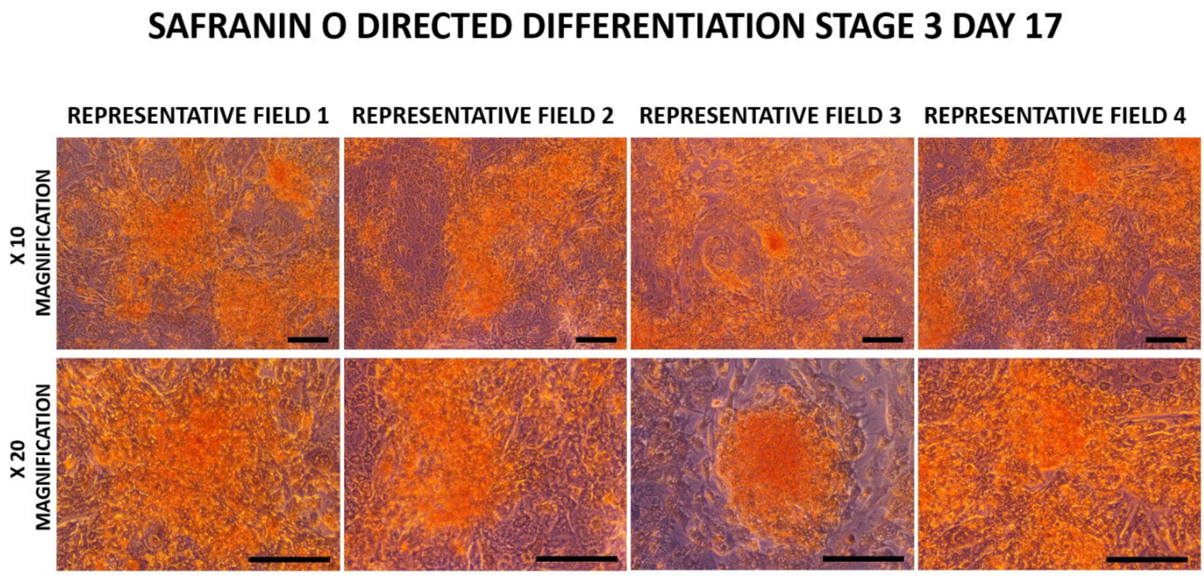

Supplemental Figure S2. Safranin O staining of UKKi26a-chondrogenic cells at Stage 3 / day 17 of directed differentiation (DD). Representative images of DD cultures at Stage 3/ day 17 were positive for extracellular matrix (ECM)-specific staining with safranin O, indicative of the presence of highly-sulphated proteoglycans. Scale bars = 50μm.

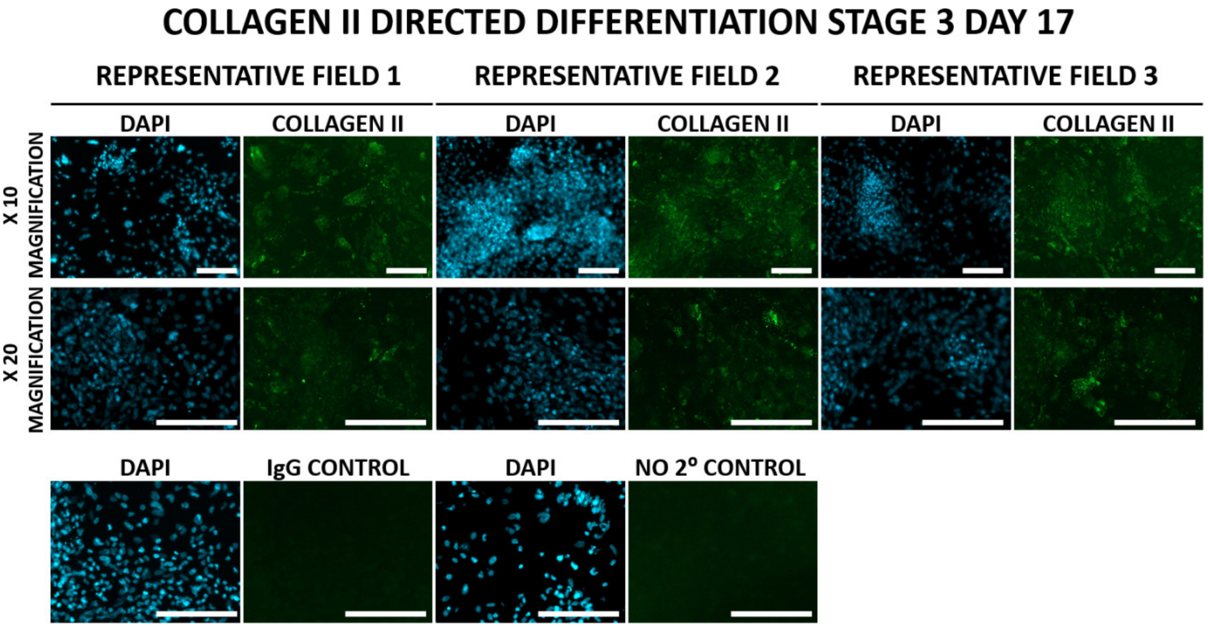

Supplemental Figure S3. Collagen type II immunolabelling of UKKi26a-chondrogenic cells at Stage 3 / day 17 of directed differentiation (DD). Representative images of DD cultures at Stage 3 / day 17 showed positive deposition of cartilage-specific collagen type II within the extracellular matrix (ECM) of cultures. Collagen type II immunolabelling (green) was counter-stained with 4′,6-diamidino-2-phenylindole (DAPI) (DAPI; blue) to identify cell nuclei and serve as a reference to collagen type II deposition within the ECM. Controls for immunolabelling reactions were performed in parallel as a primary IgG isotype control and a no secondary antibody control. Scale bars = 50µm.

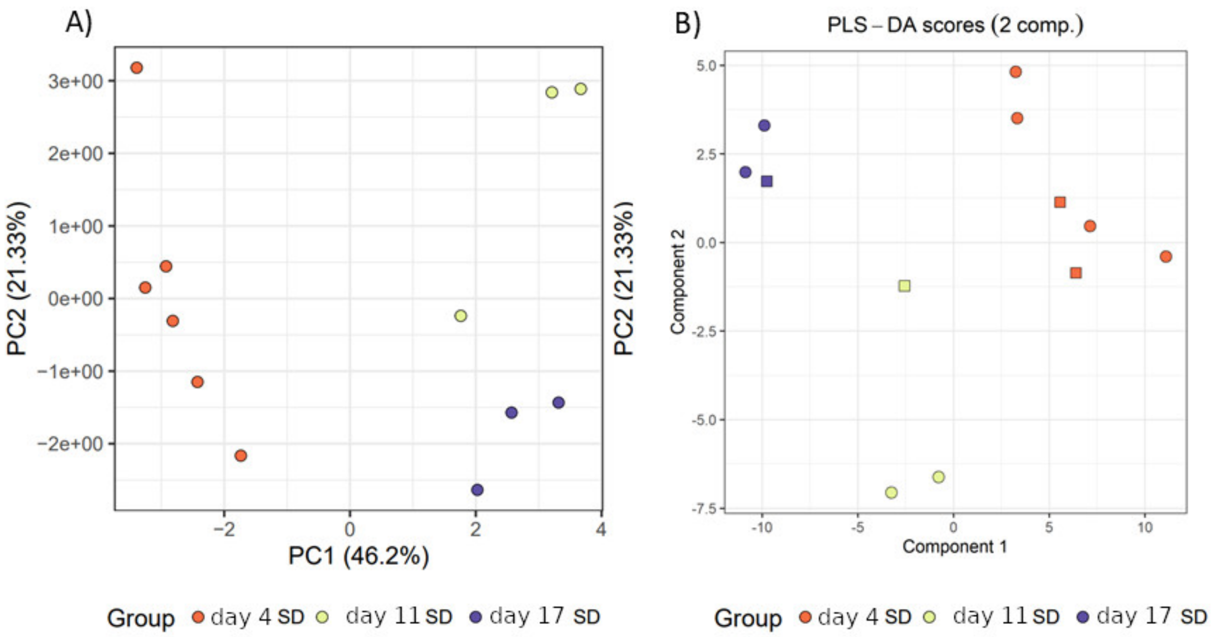

Supplemental Figure S4. PCA of extracellular metabolite profiles spontaneously differentiating cells at day 4 (equivalent to stage 1; n=6), day 11 (equivalent to stage 2; n=3) and day 17 (equivalent to stage 3; n=3). Circles or squares indicate the split of data between training and testing, respectively, for predictive PLS-DA models.

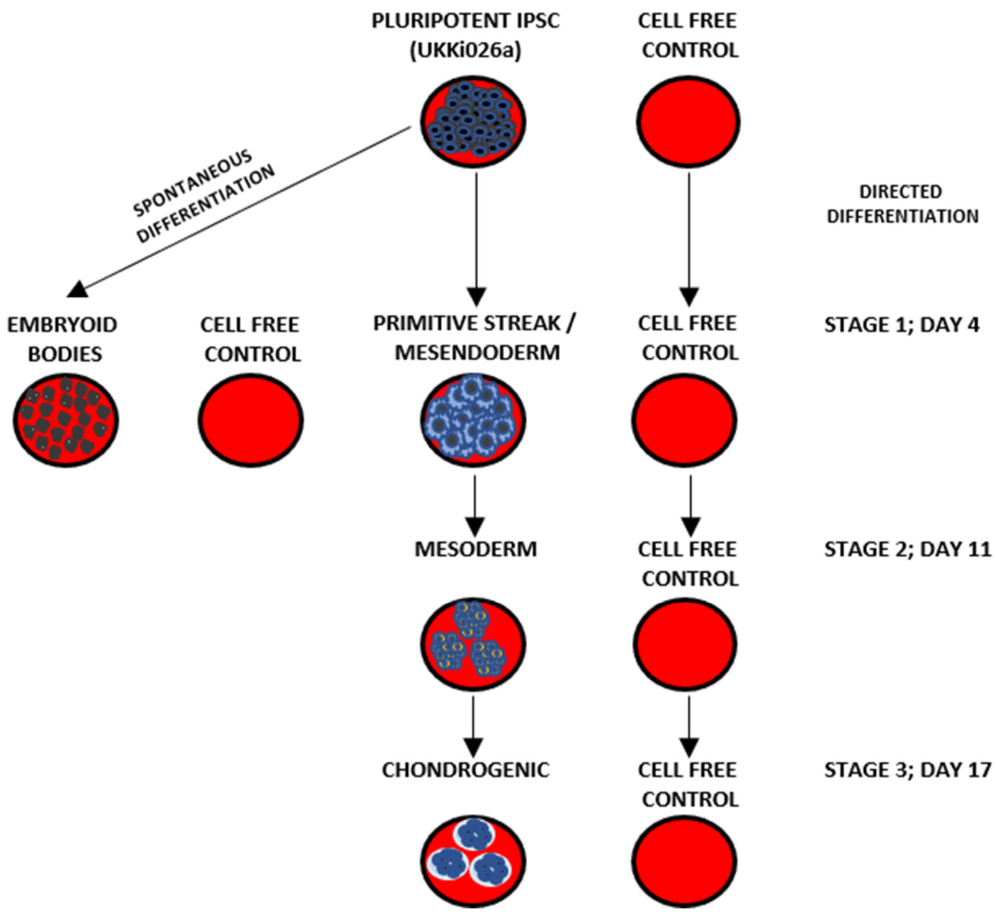

Supplemental Figure S5. Schema showing experimental design of cell culture process including differentiation of pluripotent IPSC and cell free controls. Pluripotent IPSC line UKKi026a was cultured as a homogeneous monolayer culture. Directed differentiation (DD) of pluripotent IPSC was performed by culture in a chemically-defined base medium temporally supplemented with combinations of growth factors and serial passage on defined matrix substrates (Supplemental Table 1). Comparative analysis between DD and spontaneous differentiation was performed by establishing parallel cultures of embryoid bodies from the same culture of IPSCs. Cell free controls consisting of the exact same culture conditions through differentiation pathways were established in parallel to ensure that any background molecular signature arising from plastics, matrix and media was constant across all experiments. Cell cultures and cell free controls were taken for analysis as follows: pluripotent IPSC; DD Stage 1 (primitive streak/ mesendoderm), day 4; DD Stage 2 (mesoderm), day 11; DD Stage 3 (chondrogenic cells) day 17; spontaneous differentiation (embryoid bodies).

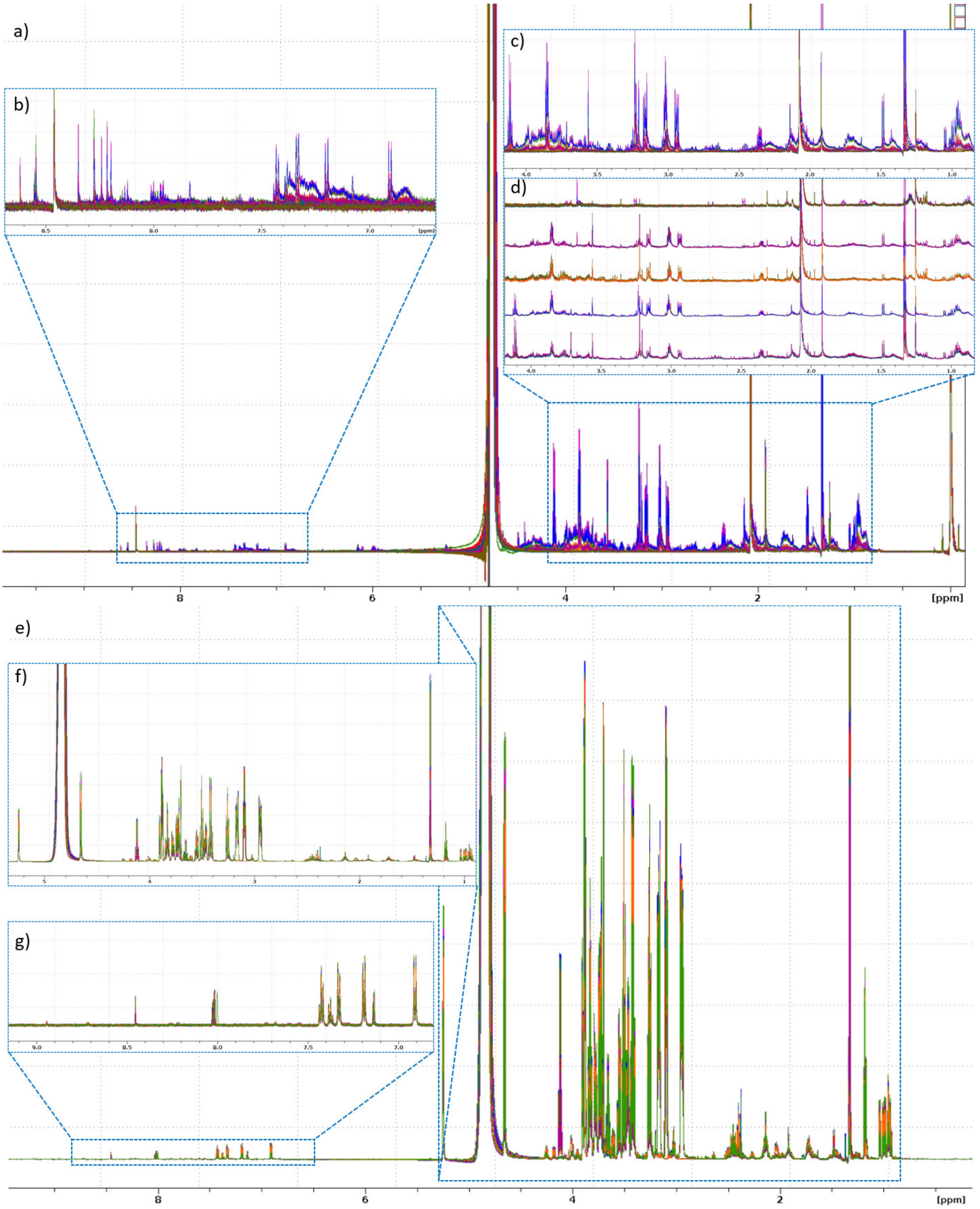

Supplemental Figure S6. Representative ^{1}H NMR Spectra of Intracellular and Extracellular Metabolites top panel and inserts – a-d) cell extract NMR spectra; a) full ^{1}H NMR spectrum of entire intracellular dataset overlaid b) insert x8 magnification aromatic region (8.8-6.7ppm), c) insert of aliphatic region (5.3-0.9ppm), d) insert of aliphatic region -stacked for each group (bottom-to-top; IPSCs, DD stage1, DD stage2, DD stage3, SD (all stages). e-g) cell media NMR spectra; e) full ^{1}H NMR spectrum of entire intracellular dataset overlaid f) insert x8 magnification aromatic region (8.8-6.7ppm), g) insert of aliphatic region (5.3-0.9 ppm).

a)

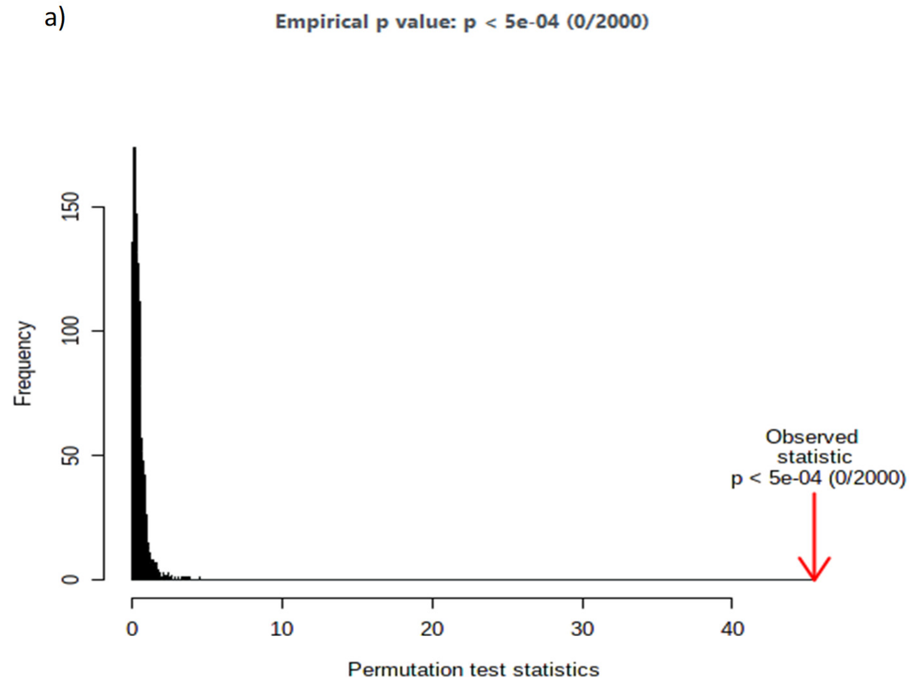
Empirical p value: p < 5e-04 (0/2000)
Frequency
0
50
100
150
Observed
statistic
p < 5e-04 (0/2000)
0
10
20
30
40
Permutation test statistics

b)

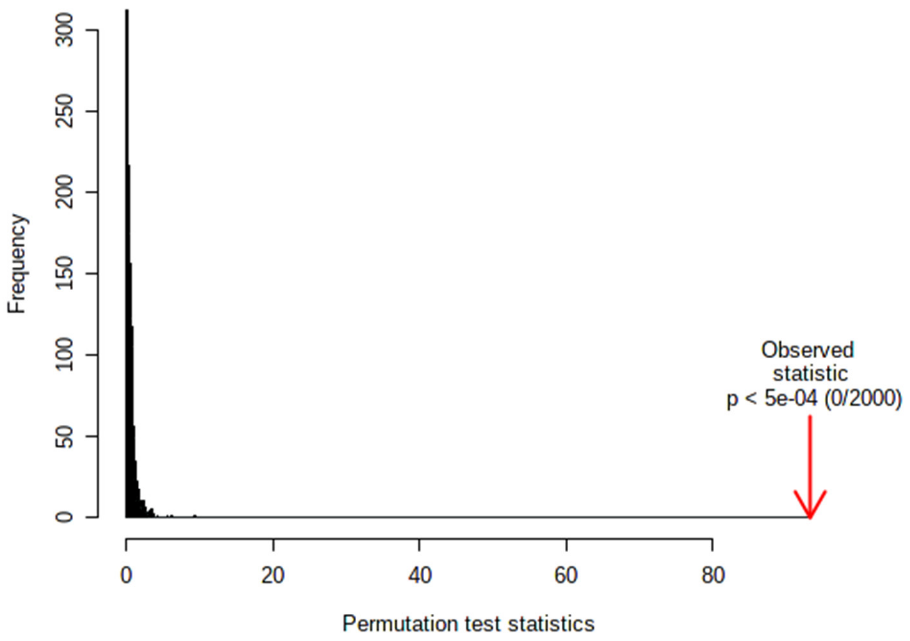
Frequency
0
50
100
150
200
250
300
Observed
statistic
p < 5e-04 (0/2000)
0
20
40
60
80
Permutation test statistics

c)

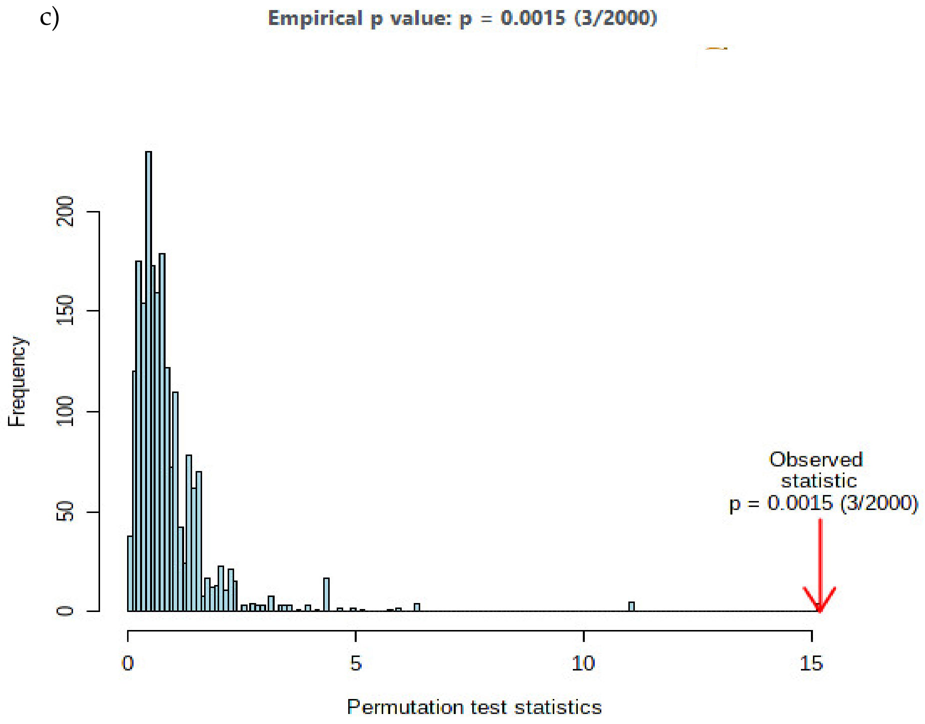

d)

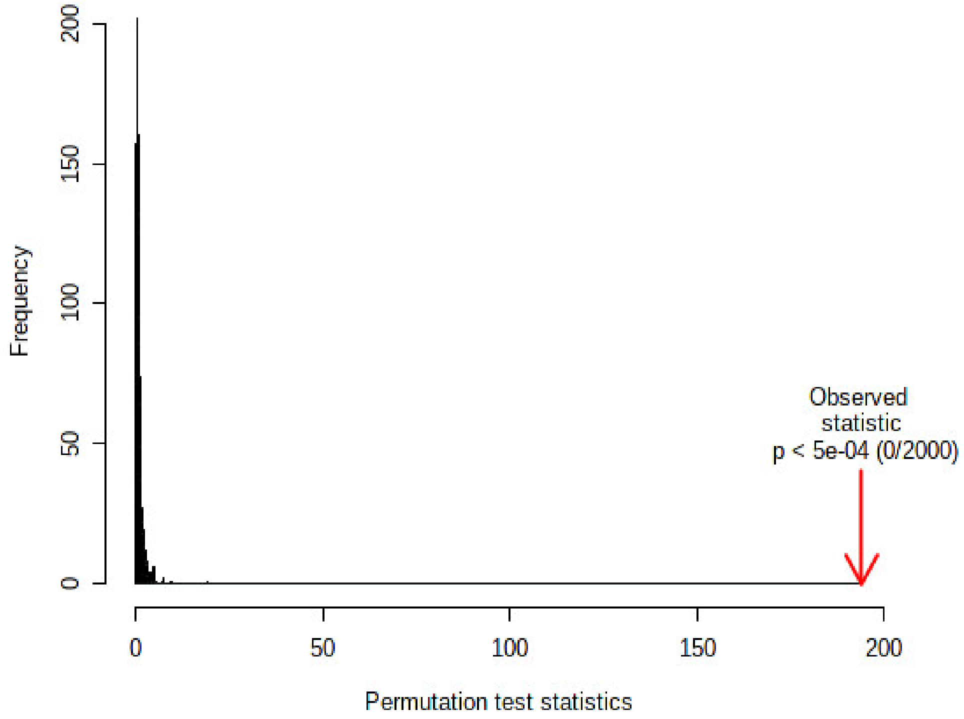

Supplemental Figure S7. Validation of PLS-DA scores PLS-DA models were validated using receiver operator characteristic (ROC) scores and 2000 permutations per model. Supplemental Figure 7a) PLS-DA Permutation testing Intracellular IPSC, DD-S1, DD-S2, DD-S3 (complements Figure 1c,. Supplemental Figure 7b) PLS-DA Permutation testing Extracellular IPSC, DD-S1, SD-S1 (complements Figure 1 d). Supplemental Figure 7c) PLS-DA Permutation testing Intracellular IPSC, DD-S1, SD-S1 (complements Figure 3 c). Supplemental Figure 7d) PLS-DA Permutation testing Extracellular IPSC, DD-S1, DD-S2, DD-S3 (complements Figure 3 d).

Supplemental Table S1. Metabolite Annotations for Intra and Extracellular Metabolic Profiles. Metabolites denoted with asterisks are the subset of methyl containing (high sensitivity) metabolites monitored in the spontaneous differentiation.

Intracellular Extracts			Extracellular Extracts		
**Metabolite Name**	**HMDB code**	**KEGG**	**Metabolite Name**	**HMDB code**	**KEGG**
**2-Aminobutyrate***	HMDB0000650	C02261	**5-Aminolevulinate**	HMDB0001149	C00430
**2-Hydroxybutyrate**	HMDB0000008	C05984	**Acetate**	HMDB0000042	C00033
**2-Methylglutarate***	HMDB0000422	NA	**Dimethylamine**	HMDB0000087	C00543
**Acetate***	HMDB0000042	C00033	**Citrate**	HMDB0000094	C00158
**Acetone**	HMDB0001659	C00207	**Choline**	HMDB0000097	C00114
**Acetylcholine**	HMDB0000895	C01996	**Ethanol**	HMDB0000108	C00469
**Adenosine**	HMDB0000050	C00212	**Glucose**	HMDB0000122	C00221
**Adenylhomocysteine**	HMDB0000939	C00021	**Glycine**	HMDB0000123	C00037
**Alanine***	HMDB0000161	C00041	**Glutathione**	HMDB0000125	NA
**Alloleucine***	HMDB0000687	C00123	**Formate**	HMDB0000142	C00058
**ATP**	HMDB0000538	C00002	**Glutamate**	HMDB0000148	C00025
**Citrate**	HMDB0000094	C00158	**Tyrosine**	HMDB0000158	C00082
**Creatine phosphate**	HMDB0001511	C02305	**Phenylalanine**	HMDB0000159	C00079
**Creatinine**	HMDB0000562	C00791	**Alanine**	HMDB0000161	C00041
**Deoxyadenosine**	HMDB0000101	C00559	**Proline**	HMDB0000162	C00148
**Dimethylamine**	HMDB0000087	C00543	**Threonine**	HMDB0000167	C00188
**Ethanol***	HMDB0000108	C00469	**Isoleucine**	HMDB0000172	C00407
**Folate**	HMDB0000121	C00504	**Histidine**	HMDB0000177	C00135
**Formate**	HMDB0000142	C00058	**Lysine**	HMDB0000182	C00047
**Glucose**	HMDB0000122	C00221	**Serine**	HMDB0000187	C00065
**Glucose-6-phosphate**	HMDB0001401	C00092	**Lactate**	HMDB0000190	C00186
**Glutamate**	HMDB0000148	C00025	**Aspartate**	HMDB0000191	C00049
**Glutamine**	HMDB0000641	C00064	**Cysteine**	HMDB0000192	C00491
**Glycerophosphocholine**	HMDB0000086	C00670	**Myo inositol**	HMDB0000211	C00137
**Glycine**	HMDB0000123	C00037	**Pyruvate**	HMDB0000243	C00022
**Histamine**	HMDB0000870	C00388	**Pyroglutamate**	HMDB0000267	C01879
**Histidine**	HMDB0000177	C00135	**Arginine**	HMDB0000517	C00062
**Homocysteine**	HMDB0000676	C01817	**Creatine**	HMDB0000562	C00791
**Hydroxyvalerate***	HMDB0000754	C20827	**Glutamine**	HMDB0000641	C00064
**Indoleacetic acid**	HMDB0000197	C00954	**2-Aminobutyrate**	HMDB0000650	C02261
**Inosine**	HMDB0000195	C00294	**Fructose**	HMDB0000660	C02336
**Inosinic acid**	HMDB0000175	C00130	**Leucine**	HMDB0000687	C00123
**Isoleucine***	HMDB0000172	C00407	**Methionine**	HMDB0000696	C00073
**Ketoleucine***	HMDB0000695	C00233	**Homocysteine**	HMDB0000742	C00155
**Lactate***	HMDB0000190	C00186	**Oxypurinol**	HMDB0000786	C07599
**Leucine***	HMDB0000557	NA	**Valine**	HMDB0000883	C00183

**Lysine***	HMDB0000182	C00047	**Acetylcholine**	HMDB0000895	C01996
**Malonate**	HMDB0000691	C04025	**NAD+**	HMDB0000902	C00003
**Methionine**	HMDB0000696	C00073	**Trimethylamine-N-oxide**	HMDB0000925	C01104
**Methylamine**	HMDB0000164	C00218	**Threonate**	HMDB0000943	C01620
**Methylhistidine**	HMDB0000001	C01152	**Phosphocreatine**	HMDB0001511	C02305
**Myo-insitol**	HMDB0000211	C00137	**Methylguanidine**	HMDB0001522	C02294
**NAD+**	HMDB0000902	C00003	**Dimethylxanthine**	HMDB0001860	C13747
**NADP+**	HMDB0000217	C00006	**Methanol**	HMDB0001875	C00132
**Niacinamide**	HMDB0001406	C00153	**Dimethylsulphate**	HMDB0002303	C00580
**Oxypurinol**	HMDB0000786	C07599	**Dimethylurate**	HMDB0011103	C16356
**Phenylacetate**	HMDB0000209	C07086			
**phenylalanine**	HMDB0000159	C00079			
**Pyroglutamate**	HMDB0000267	C01879			
**Pyruvate**	HMDB0000243	C00022			
**Succinate**	HMDB0000254	C00042			
**Sucrose**	HMDB0000258	C00089			
**Taurine**	HMDB0000251	C00245			
**Threonine**	HMDB0000167	C00188			
**Tyrosine**	HMDB0000158	C00082			
**Valine***	HMDB0000883	C00183			
**Xanthine**	HMDB0000292	C00385			

Supplemental Table S2. Chemical shift variable boundaries for metabolite binning.

Extracellular Bin Boundary/Metabolite Annotation				Intracellular Bin Boundary/Metabolite Annotation			
Database Identifier	Metabolite Identification	Chemical Shift (ppm)	Overlap	Database Identifier	Metabolite Identification	Chemical Shift (ppm)	Overlap
		[8.9579…8.9298]				[8.9514…8.9338]	
		[8.7354…8.7004]		HMDB0001406	Niacinamide	[8.7327…8.71]	
HMDB0000142	Formic acid	[8.4632…8.4401]				[8.6241…8.6042]	
HMDB0000786	Oxypurinol	[8.2752…8.2422]		HMDB0000175	Inosinic acid	[8.558…8.5276]	
HMDB0000902	NAD	[8.2289…8.1938]		HMDB0000142	Formic acid	[8.4678…8.446]	
HMDB0000177	L-Histidine	[8.0037…7.9876]		HMDB0000101	Deoxyadenosine	[8.3558…8.335]	
		[7.9114…7.9002]		HMDB0000538	Adenosine triphosphate	[8.3219…8.3125]	
		[7.7411…7.7142]		HMDB0000786	Oxypurinol	[8.2804…8.2661]	
		[7.6858…7.6694]		HMDB0000050	Adenosine	[8.2465…8.2348]	
		[7.6196…7.5793]		HMDB0000902	NAD	[8.2199…8.2056]	
		[7.5637…7.5308]		HMDB0000217	NADP	[8.2028…8.1904]	
HMDB0000159	L-Phenylalanine	[7.4429…7.4076]				[8.1652…8.11]	
HMDB0000159	L-Phenylalanine	[7.3891…7.3575]				[8.0186…8.0083]	
HMDB0000159	L-Phenylalanine	[7.3442…7.3091]		HMDB0000001	1-Methylhistidine	[8.0076…8.0041]	
		[7.2986…7.2614]		HMDB0000939	S-Adenosylhomocysteine	[8.0004…7.9744]	
HMDB0000158	L-Tyrosine	[7.2067…7.1716]		HMDB0000177	L-Histidine	[7.9743…7.9444]	overlapped with HMDB0000195
HMDB0000177	L-Histidine	[7.1464…7.12]		HMDB0000292	Xanthine	[7.8891…7.8665]	
HMDB0000158	L-Tyrosine	[6.9232…6.892]		HMDB0000870	Histamine	[7.8476…7.825]	
HMDB0000122	D-Glucose	[5.2626…5.2228]				[7.6883…7.6737]	
HMDB0000167	L-Threonine	[4.2713…4.2123]		HMDB0000121	Folic acid	[7.6118…7.586]	
HMDB0000267	Pyroglutamic acid	[4.1979…4.1576]				[7.5564…7.5331]	

HMDB0001149	5-Aminolevulinic acid	[4.1499...4.142]		HMDB0000159	L-Phenylalanine	[7.452...7.4174]	
HMDB0000190	L-Lactic acid	[4.1376...4.0975]	overlapped with HMDB0000192, HMDB0000162	HMDB0000159	L-Phenylalanine	[7.3959...7.3772]	
HMDB0000660	D-Fructose	[4.095...4.0877]				[7.3958...7.3646]	
HMDB0000211	Myoinositol	[4.0857...4.055]		HMDB0000159	L-Phenylalanine	[7.3466...7.323]	
HMDB0000562	Creatinine	[4.0549...4.048]				[7.319...7.2135]	
HMDB0000660	D-Fructose	[4.0367...4.0276]		HMDB0000158	L-Tyrosine	[7.2093...7.1882]	
HMDB0000660	D-Fructose	[4.0194...4.0118]				[7.1841...7.09]	
HMDB0000187	L-Serine	[4.0116...4.0045]	overlapped with HMDB0000943	HMDB0000001	1-Methylhistidine	[7.0872...7.0714]	
HMDB0000660	D-Fructose	[4.0018...3.9787]	overlapped with HMDB0000159, HMDB0000177	HMDB0000158	L-Tyrosine	[6.9179...6.892]	
		[3.9683...3.9622]				[6.8737...6.797]	
HMDB0001511	Phosphocreatine	[3.9619...3.9424]				[6.5249...6.5192]	
HMDB0000158	L-Tyrosine	[3.9422...3.9171]	overlapped with HMDB0000192	HMDB0000538	Adenosine triphosphate	[6.1629...6.1449]	overlapped with HMDB0000175
HMDB0000191	L-Aspartic acid	[3.9116...3.9043]		HMDB0000195	Inosine	[6.1148...6.0973]	
HMDB0000122	D-Glucose	[3.9006...3.8934]	overlapped with HMDB0000191, HMDB0000660, HMDB0000742, HMDB0000317			[6.01...5.9989]	
HMDB0000660	D-Fructose	[3.892...3.8808]	overlapped with HMDB0000742,			[5.9987...5.9669]	

			HMDB0000191, HMDB0000317				
HMDB0000122	D-Glucose	[3.8805...3.859]	overlapped with HMDB0000660, HMDB0000742			[4.4482...4.4257]	
HMDB0000696	L-Methionine	[3.8613...3.8541]				[4.4256...4.4162]	
HMDB0000122	D-Glucose	[3.8522...3.84]	overlapped with HMDB0000696			[4.4095...4.393]	
HMDB0000122	D-Glucose	[3.8398...3.8106]				[4.3929...4.3715]	
HMDB0000161	L-Alanine	[3.8055...3.7958]		HMDB0000086	Glycerophosphocholine	[4.3508...4.267]	
HMDB0000517	L-Arginine	[3.7047...3.7793]	overlapped with HMDB0000660	HMDB0000167	L-Threonine	[4.2666...4.2287]	
HMDB0000161	L-Alanine	[3.7792...3.7703]	overlapped with HMDB0000660, HMDB0000122, HMDB0000641,	HMDB0000267	Pyroglutamic acid	[4.2006...4.1525]	
HMDB0000641	L-Glutamine	[3.7702...3.7609]				[4.1524...4.1424]	
HMDB0000641	L-Glutamine	[3.7608...3.752]	overlapped with HMDB0000161, HMDB0000122	HMDB0000190	L-Lactic acid	[4.1385...4.0893]	
		[3.7519...3.7454]		HMDB0000562	Creatinine	[4.082...4.0712]	
HMDB0000122	D-Glucose	[3.7443...3.7145]	overlapped with HMDB0000517	HMDB0000211	Myoinositol	[4.0711...4.0506]	
HMDB0000122	D-Glucose	[3.7121...3.6933]	overlapped with HMDB0000660	HMDB0000863	Isopropyl alcohol	[4.0505...3.9985]	
HMDB0000943	Threonic acid	[3.69...3.6797]		HMDB0000211	Myoinositol	[3.9984...3.9756]	

HMDB0000943	Threonic acid	[3.6827...3.6763]	overlapped with HMDB0000172, HMDB0000660	HMDB0001511	Phosphocreatine	[3.9737...3.9233]	overlapped with HMDB0000159
		[3.6762...3.6668]		HMDB00000122		[3.923...3.9008]	
HMDB0000943	Threonic acid	[3.6646...3.6453]	overlapped with HMDB0000660, HMDB0000108	HMDB0000676	L-Homocystine	[3.9007...3.8707]	overlapped with HMDB00000122
HMDB0000211	Myoinositol	[3.645...3.6374]	overlapped with HMDB0000943	HMDB0000258	Sucrose	[3.8706...3.8406]	overlapped with HMDB0000012, HMDB0000740
HMDB0000211	Myoinositol	[3.6321...3.6015]	overlapped with HMDB0000883			[3.8396...3.8316]	
HMDB0000167	L-Threonine	[3.6041...3.59]				[3.831...3.8261]	
HMDB0000660	D-Fructose	[3.572...3.5636]				[3.826...3.819]	
HMDB0000123	Glycine	[3.5609...3.5562]		HMDB0000740	Lactulose	[3.817...3.7981]	overlapped with HMDB00000122
HMDB0000122	D-Glucose	[3.5548...3.5489]	overlapped with HMDB0000211	HMDB0000740	Lactulose	[3.7972...3.7546]	overlapped with HMDB0000012, HMDB0000182
HMDB0000122	D-Glucose	[3.5462...3.5344]	overlapped with HMDB0000660	HMDB0000211	Myoinositol	[3.7532...3.7361]	overlapped with HMDB0000012, HMDB0000182, HMDB0000172
HMDB0000122	D-Glucose	[3.5296...3.5232]	overlapped with HMDB0000211	HMDB00000023		[3.7349...3.6987]	overlapped with HMDB0000012, HMDB0000211

HMDB0000122	D-Glucose	[3.5206...3.3885]		HMDB0000258	Sucrose	[3.6986...3.6599]	overlapped with HMDB0000012, HMDB0000211
HMDB0000192	L-Cystine	[3.3909...3.382]		HMDB0000197	Indoleacetic acid	[3.6598...3.6015]	overlapped with HMDB0000012, HMDB0000211
HMDB0000162	L-Proline	[3.377...3.364]		HMDB0000167	L-Threonine	[3.6014...3.5872]	
HMDB0000192	L-Cystine	[3.3637...3.3541]	overlapped with HMDB0000162			[3.5863...3.5653]	
HMDB0001875	Methanol	[3.3534...3.3465]		HMDB0000123	Glycine	[3.5652...3.5545]	overlapped with HMDB00000122
HMDB0000162	L-Proline	[3.3459...3.3421]		HMDB00000122		[3.5536...3.521]	
HMDB0000162	L-Proline	[3.342...3.338]		HMDB0000209	Phenylacetic acid	[3.5207...3.5143]	overlapped with HMDB00000023
HMDB0000162	L-Proline	[3.3362...3.3305]		HMDB0001401	Glucose 6-phosphate	[3.5125...3.5046]	overlapped with HMDB00000122
HMDB0001860	Paraxanthine	[3.3278...3.3203]				[3.5045...3.4998]	
HMDB0011103	1,7-Dimethyluric acid	[3.3202...3.3145]		HMDB0001401	Glucose 6-phosphate	[3.4991...3.4924]	overlapped with HMDB00000122
HMDB0000211	Myoinositol	[3.3121...3.3028]		HMDB00000122		[3.4906...3.478]	
HMDB0000159	L-Phenylalanine	[3.3015...3.296]		HMDB00000122		[3.4773...3.455]	
HMDB0000211	Myoinositol	[3.2959...3.2905]	overlapped with HMDB0000159			[3.4518...3.44]	
HMDB0000122	D-Glucose	[3.2754...3.645]	overlapped with HMDB0000211, HMDB0000159			[3.435...3.376]	

HMDB0000122	D-Glucose	[3.2625...3.2506]	overlapped with HMDB0000177	HMDB0000001	1-Methylhistidine	[3.3639...3.3556]	
HMDB0000925	Trimethylamine N-oxide	[3.2482...3.2403]	overlapped with HMDB0000517, HMDB0000177	HMDB0000211	Myoinositol	[3.305...3.2662]	
HMDB0000517	L-Arginine	[3.2396...3.2319]	overlapped with HMDB0000177, HMDB0000192	HMDB0000159	L-Phenylalanine	[3.2659...3.244]	
HMDB0000192	L-Cystine	[3.2296...3.2207]	overlapped with HMDB0000158, HMDB0000517	HMDB00000122		[3.242...3.228]	
HMDB0000895	Acetylcholine	[3.2135...3.2038]		HMDB0000251	Taurine	[3.2278...3.2194]	overlapped with HMDB00000122
HMDB0000192	L-Cystine	[3.2038...3.196]		HMDB0000086	Glycerophosphocholine	[3.2178...3.2141]	
HMDB0000097	Choline	[3.1943...3.1855]	overlapped with HMDB0000192, HMDB0000158	HMDB0000895	Acetylcholine	[3.2118...3.2028]	
HMDB0000158	L-Tyrosine	[3.1845...3.1783]				[3.1923...3.1854]	
HMDB0000192	L-Cystine	[3.1782...3.1737]				[3.1839...3.1417]	
HMDB0000177	L-Histidine	[3.1736...3.153]				[3.1414...3.135]	
HMDB0002303	Dimethylsulfide	[3.1485...3.1432]		HMDB0000691	Malonic acid	[3.1334...3.1267]	
HMDB0000177	L-Histidine	[3.1388...3.1335]	overlapped with HMDB0000159			[3.1262...3.1214]	
HMDB0000177	L-Histidine	[3.1334...3.1267]	overlapped with HMDB0000159	HMDB0000159	L-Phenylalanine	[3.1213...3.0967]	
HMDB0000159	L-Phenylalanine	[3.1266...3.1214]				[3.0965...3.0113]	

HMDB0000158	L-Tyrosine	[3.0827...3.0747]		HMDB0000182	L-Lysine	[3.0112...2.9715]	
HMDB0000158	L-Tyrosine	[3.0716...3.0639]	overlapped with HMDB0000192			[2.9714...2.9193]	
HMDB0000192	L-Cystine	[3.0611...3.054]				[2.886...2.864]	
HMDB0000158	L-Tyrosine	[3.0553...3.0534]	overlapped with HMDB0000192			[2.8625...2.8477]	
HMDB0000192	L-Cystine	[3.0528...3.0497]				[2.8466...2.8206]	
HMDB0000562	Creatinine	[3.0491...3.043]	overlapped with HMDB0000158			[2.811...2.7965]	
HMDB0000182	L-Lysine	[3.0429...3.0371]				[2.7964...2.764]	
HMDB0001511	Phosphocreatine	[3.037...3.0302]				[2.7626...2.754]	
HMDB0000562	Creatinine	[3.0284...3.0188]	overlapped with HMDB0000182	HMDB0000087	Dimethylamine	[2.7379...2.7298]	
HMDB0000182	L-Lysine	[3.0176...3.0091]		HMDB0000094	Citric acid	[2.7297...2.6938]	
		[3.0088...2.9808]		HMDB0000696	L-Methionine	[2.6937...2.6683]	overlapped with HMDB0000164
HMDB0000125	Glutathione	[2.9676...2.9557]		HMDB0000164	Methylamine	[2.6681...2.6332]	overlapped with HMDB0000696
HMDB0000125	Glutathione	[2.9548...2.9403]		HMDB0000696	L-Methionine	[2.6326...2.5984]	overlapped with HMDB0000164
HMDB0000125	Glutathione	[2.9389...2.9308]		HMDB0000267	Pyroglutamic acid	[2.5983...2.5349]	
HMDB0000125	Glutathione	[2.9122...2.889]		HMDB0000641	L-Glutamine	[2.5348...2.4347]	
		[2.8827...2.8629]		HMDB0000254	Succinic acid	[2.4229...2.4138]	
HMDB0001522	Methylguanidine	[2.8614...2.8535]		HMDB0000243	Pyruvic acid	[2.4094...2.3989]	
HMDB0000191	L-Aspartic acid	[2.8532...2.8311]		HMDB0000883	L-Valine	[2.3983...2.3873]	
HMDB0000191	L-Aspartic acid	[2.8233...2.8087]		HMDB0000883	L-Valine	[2.3835...2.3244]	

		[2.8001...2.7874]				[2.3236...2.3098]	
HMDB0000087	Dimethylamine	[2.7612...2.7515]		HMDB0001659	Acetone	[2.3097...2.2432]	overlapped with HMDB0000883
HMDB0000191	L-Aspartic acid	[2.7269...2.7052]				[2.2431...2.2295]	
HMDB0000094	Citric acid	[2.6977...2.6916]		HMDB0000696	L-Methionine	[2.211...2.1553]	overlapped with HMDB0000148
HMDB0000742	Homocysteine	[2.6855...2.6725]		HMDB0000148	L-Glutamic acid	[2.1538...2.1018]	overlapped with HMDB0000190
HMDB0000094	Citric acid	[2.6723...2.6565]				[2.033...2.011]	
HMDB0000742	Homocysteine	[2.6563...2.6247]	overlapped with HMDB0000696			[2.012...2.00]	
HMDB0000267	Pyroglutamic acid	[2.5528...2.543]				[1.9828...1.966]	
HMDB0001149	5-Aminolevulinic acid	[2.5374...2.5266]		HMDB0000042	Acetic acid	[1.9279...1.9021]	
HMDB0000267	Pyroglutamic acid	[2.5263...2.4803]	HMDB0001149			[1.8325...1.8198]	
HMDB0000641	L-Glutamine	[2.48...2.4698]	overlapped with HMDB0000267			[1.7998...1.788]	
HMDB0000641	L-Glutamine	[2.4682...2.4235]		HMDB0000008	2-Hydroxybutyric acid	[1.7639...1.6155]	overlapped with HMDB0000557
HMDB0000267	Pyroglutamic acid	[2.4225...2.3857]				[1.6144...1.5691]	
HMDB0000243	Pyruvic acid	[2.3832...2.3673]				[1.569...1.5032]	
HMDB0000148	L-Glutamic acid	[2.3658...2.2961]	overlapped with HMDB0000162	HMDB0000161	L-Alanine	[1.5013...1.4705]	
HMDB0000883	L-Valine	[2.2956...2.237]		HMDB0000182	L-Lysine	[1.4682...1.3544]	
HMDB0000641	L-Glutamine	[2.1942...2.0898]	overlapped with HMDB0000148	HMDB0000167	L-Threonine	[1.3432...1.3134]	overlapped with HMDB0000190

HMDB0000148	L-Glutamic acid	[2.0873...2.0045]	overlapped with HMDB0000267, HMDB0000162	HMDB0000172	L-Isoleucine	[1.3133...1.2602]	
HMDB0000162	L-Proline	[1.9982...1.9515]		HMDB0000754	3-Hydroxyisovaleric acid	[1.2594...1.2415]	
HMDB0000517	L-Arginine	[1.947...1.9214]	overlapped with HMDB0000182 HMDB0000650			[1.2366...1.2051]	
HMDB0000042	Acetic acid	[1.9212...1.9157]		HMDB0000108	Ethanol	[1.1994...1.1962]	
HMDB0000517	L-Arginine	[1.9152...1.855]	overlapped with HMDB0000182			[1.1925...1.1916]	
HMDB0000517	L-Arginine	[1.7894...1.6407]	overlapped with HMDB0000182	HMDB0000108	Ethanol	[1.1916...1.175]	
		[1.6397...1.6308]				[1.184...1.1699]	
		[1.5534...1.52]				[1.1547...1.1375]	
HMDB0000182	L-Lysine	[1.5195...1.4917]				[1.1147...1.0788]	
HMDB0000161	L-Alanine	[1.4913...1.4584]				[1.075...1.0647]	
HMDB0000172	L-Isoleucine	[1.458...1.4078]	overlapped with HMDB0000182	HMDB0000883	L-Valine	[1.0576...1.0313]	
		[1.3734...1.3656]		HMDB0000172	L-Isoleucine	[1.0238...1.0052]	
HMDB0000167	L-Threonine	[1.3441...1.3273]		HMDB0000883	L-Valine	[1.005...0.9848]	
HMDB0000190	L-Lactic acid	[1.3272...1.3205]		HMDB0000557	L-Alloisoleucine	[0.9829...0.9718]	overlapped with HMDB0000687
HMDB0000167	L-Threonine	[1.3204...1.3151]		HMDB0000557	L-Alloisoleucine	[0.9704...0.9503]	overlapped with HMDB0000172, HMDB0000650, HMDB0000008

HMDB0000190	L-Lactic acid	[1.315...1.3055]		HMDB0000650	D-Alpha-aminobutyric acid	[0.95...0.9393]	overlapped with HMDB0000172, HMDB0000695, HMDB0000008
HMDB0000172	L-Isoleucine	[1.2812...1.2247]		HMDB0000695	Ketoleucine	[0.9382...0.9267]	overlapped with HMDB0000172, HMDB0000008
HMDB0000108	Ethanol	[1.1929...1.1414]		HMDB0000422	2-Methylglutaric acid	[0.925...0.9112]	overlapped with HMDB0000008
		[1.1246...1.1079]				[0.9108...0.8831]	
		[1.1015...1.0549]				[0.8816...0.8429]	
HMDB0000883	L-Valine	[1.0444...1.0274]				[0.7848...0.7717]	
HMDB0000172	L-Isoleucine	[1.0117...0.9888]				[0.7477...0.7279]	
HMDB0000883	L-Valine	[0.9851...0.978]					
HMDB0000687	L-Leucine	[0.9696...0.9508]					
HMDB0000172	L-Isoleucine	[0.95...0.9403]	overlapped with unknown				
HMDB0000172	L-Isoleucine	[0.9381...0.9295]	overlapped with HMDB0000650 unknown				
HMDB0000650	D-Alpha-aminobutyric acid	[0.9273...0.9196]	overlapped with HMDB0000172				
HMDB0000650	D-Alpha-aminobutyric acid	[0.9116...0.9056]					
		[0.8903...0.8804]					
		[0.1646...0.1547]					

Supplemental Table S3. Variable Importance in Projection scores (VIPs) resulting from the comparison of S1DD and S1SD media samples with respect to the IPSC groups from PLS-DA models for individual (2 group comparisons). VIP scores >1 are presented alongside metabolite annotations and ANOVA univariate testing results. Significance level determined at * <0.05, ** <0.01, *** <0.001.

S1DD		S1SD	
**Metabolite Annotation**	**VIP Score**	**Metabolite Annotation**	**VIP Score**
Formate***	1.218473	Arginine***	1.11094
Glutathione	1.213458	Lactate***	1.110872
Threonine***	1.212801	Lysine***	1.109737
Phenylalanine***	1.211109	Glutamine***	1.109512
Pyruvate***	1.210975	Proline***	1.108974
Glycine***	1.209096	Creatine***	1.10893
Aspartate**	1.206322	Phosphocreatine***	1.108904
Glutamine***	1.205866	Valine***	1.107641
Glucose*	1.203588	Glutamate***	1.107198
Histidine***	1.202707	Threonine***	1.106748
Glutamate***	1.20054	Glucose**	1.104057
Cysteine***	1.195824	Isoleucine***	1.103927
Pyroglutamate***	1.188956	Formate***	1.103476
Alanine***	1.188393	Glycine***	1.102678
Acetate***	1.184197	2 Aminobutyrate***	1.101023
Methionine***	1.167963	Leucine***	1.10001
Creatine***	1.155498	Acetate***	1.098555
Proline***	1.152625	Phenylalanine***	1.096797
Lactate***	1.14968	Myo inositol***	1.0961
Fructose***	1.14696	Cysteine***	1.092953
Threonate***	1.141046	Tyrosine***	1.09241
Choline***	1.050961	Methanol	1.09195
Homocysteine**	1.021726	Pyruvate***	1.090073
Acetylcholine***	1.004577	Histidine***	1.083661
		Homocysteine***	1.075609
		Choline***	1.073675
		Alanine***	1.054214
		Fructose***	1.050927
		Acetylcholine***	1.046391
		Pyroglutamate***	1.032033
		Methionine***	1.008401
		Serine***	1.007691
		Glutathione***	1.004739

Supplemental Table S4. MSEA for S1DD vs IPSCs Extracellular Metabolites. Significantly enriched pathways ($p < 0.05$) are presented with their component metabolite annotations. ANOVA significant metabolites vs their respective cell-free controls are coloured blue (decreased vs IPSCs), red (increased vs IPSCs) or black (non-significant in ANOVA).

Metabolic Pathway	Metabolite Annotations	P value	FDR
Glycine and Serine	Creatine, Glycine, Glutamate, Alanine, Threonine, Pyruvate,	2.52E-06	1.80E-04
Glutathione Metabolism	Glycine, Glutathione, Glutamate, Alanine, Pyroglutamate,	3.68E-06	1.80E-04
Glutamate Metabolism	Glycine, Glutathione, Glutamate, Alanine, Aspartate, Pyruvate,	6.33E-06	2.07E-04
Ammonia Recycling	Glycine, Glutamate, Histidine, Aspartate, Pyruvate, Glutamine	5.18E-05	0.00127
Glucose-Alanine Cycle	Glucose, Glutamate, Alanine, Pyruvate	1.45E-04	0.00284
Urea Cycle	Glutamate, Alanine, Aspartate, Pyruvate, Glutamine	3.72E-04	0.00608
Alanine Metabolism	Glycine, Glutamate, Alanine, Pyruvate	4.52E-04	0.00633
Amino Sugar	Acetate, Glutamate, Pyruvate, Glutamine, Fructose	6.99E-04	0.00856
Methionine Metabolism	Choline, Glycine, Cysteine, Methionine, Homocysteine	0.00242	0.0263
Arginine and Proline	Creatine, Glycine, Glutamate, Proline, Aspartate	0.00615	0.0603
Aspartate Metabolism	Acetate, Glutamate, Aspartate, Glutamine	0.00748	0.0666
Warburg Effect	Glucose, Glutamate, Lactate, Pyruvate, Glutamine	0.00907	0.0741
Betaine Metabolism	Choline, Methionine, Homocysteine	0.0114	0.086
Homocysteine	Cysteine, Homocysteine	0.0172	0.12
Cysteine Metabolism	Glutamate, Pyruvate, Cysteine	0.0207	0.122
Malate-Aspartate	Glutamate, Aspartate	0.0211	0.122
Pyruvaldehyde	Glutathione, Pyruvate	0.0211	0.122
Pyruvate Metabolism	Acetate, Glutathione, Lactate, Pyruvate	0.0226	0.123
Beta-Alanine	Glutamate, Histidine, Aspartate	0.042	0.217
Gluconeogenesis	Glucose, Lactate, Pyruvate	0.0453	0.222

Supplemental Table S5. One-way ANOVA with Tukeys post hoc analysis and Variable Importance in Projection scores (VIPs) Resulting from the Four-Group Comparison of DD Samples with Respect to the IPSC groups from PLS-DA Models for Intracellular and Media Datasets. VIP scores >1 are presented alongside metabolite annotations.

**Intracellular Dataset**		**Media Dataset**	
**Metabolite Annotation**	**VIP Score**	**Metabolite Annotation**	**VIP Score**
Sucrose	1.723663	Proline	2.038509
2-Methylglutarate	1.688617	Methanol	1.697869
Glucose-6-phosphate	1.595152	Threonate	1.647413
Acetylcholine	1.483247	Aspartate	1.632209
Oxypurinol	1.481848	Acetylcholine	1.628173
Glycine	1.435978	Dimethylxanthine	1.620004
Ketoleucine	1.433853	Choline	1.575335
Tyrosine	1.430902	Homocysteine	1.545205
Adenosine	1.42446	Acetate	1.46985
Deoxyadenosine	1.378853	Formate	1.359794
phenylalanine	1.28141	2 Aminobutyrate	1.198795
Homocysteine	1.257761	Glutathione	1.061053
Lysine	1.236632	Dimethylurate	1.060311
2-Aminobutyrate	1.202638	Leucine	1.02976
Creatine phosphate	1.192762		
2-Hydroxybutyrate	1.179124		
Pyroglutamate	1.139851		
Histidine	1.12535		
Xanthine	1.109243		
Inosinic acid	1.08561		
Pyruvate	1.065754		
Methionine	1.065006		
actate	1.000463		

Metabolite Annotation	Metabolite HMDB ID	one-way adjusted p-	Tukey's post hoc analysis					
			S1-	S2-	S3-	S2-S1	S3-S1	S3-S2
2-Aminobutyrate	HMDB0000650	0.0001	0.9862	0.0001	0.9955	0.0001	0.9995	0.0001
2-	HMDB0000008	0.0000	1.0000	0.0000	0.9976	0.0000	0.9987	0.0000
2-Methylglutarate	HMDB0000422	0.0005	0.1648	0.0015	0.9239	0.0966	0.0394	0.0003
Acetate	HMDB0000042	0.0000	0.0037	0.0000	0.6532	0.0000	0.0002	0.0000
Acetone	HMDB0001659	0.0000	0.8039	0.0000	0.0267	0.0000	0.1254	0.0015
Acetylcholine	HMDB0000895	0.0000	0.0137	0.0000	0.0000	0.0000	0.0000	0.0016
Adenosine	HMDB0000050	0.0061	0.5331	0.0175	0.0050	0.1853	0.0671	0.9717
Alanine	HMDB0000161	0.0000	0.2036	0.0000	0.5807	0.0000	0.0121	0.0000
Citrate	HMDB0000094	0.0034	0.9627	0.0104	0.1450	0.0026	0.0455	0.4670
Creatine	HMDB0001511	0.0010	0.0009	0.0399	0.0007	0.3874	0.9984	0.3114
Deoxyadenosine	HMDB0000101	0.0093	0.4143	0.0353	0.0053	0.4282	0.1068	0.8697
Ethanol	HMDB0000108	0.0000	0.0100	0.0000	0.7591	0.0000	0.0007	0.0000
Formate	HMDB0000142	0.0000	0.0142	0.0000	0.5999	0.0000	0.0006	0.0000
Glucose	HMDB0000122	0.0196	0.0092	0.6777	0.5008	0.1001	0.1289	0.9945
Glucose-6-	HMDB0001401	0.0000	0.0001	0.0000	0.0020	0.0011	0.5757	0.0001
Glutamate	HMDB0000148	0.0003	0.9287	0.0006	0.0028	0.0015	0.0070	0.8095
Glutamine	HMDB0000641	0.0136	0.7528	0.0576	0.9868	0.0056	0.5177	0.0865
Homocysteine	HMDB0000676	0.0003	0.0001	0.0103	0.0009	0.1850	0.6286	0.7709

Hydroxyvalerate	HMDB0000754	0.0000	0.0237	0.0000	0.8916	0.0000	0.0035	0.0000
Indoleacetic acid	HMDB0000197	0.0098	0.7915	0.0048	0.4683	0.0237	0.9360	0.0723
Inosinic acid	HMDB0000175	0.0001	0.0080	0.0001	0.0000	0.1654	0.0560	0.9677
Isoleucine	HMDB0000172	0.0000	0.0002	0.0018	0.9897	0.0000	0.0002	0.0006
Ketoleucine	HMDB0000695	0.0001	0.8680	0.0002	0.8982	0.0006	0.4399	0.0000
Lactate	HMDB0000190	0.0000	0.6432	0.0001	0.0001	0.0008	0.0004	0.9995
Leucine	HMDB0000557	0.0000	0.0692	0.0012	0.5750	0.0000	0.4995	0.0001
Lysine	HMDB0000182	0.0012	0.9789	0.0030	0.9408	0.0046	0.7485	0.0006
Malonate	HMDB0000691	0.0001	0.8665	0.0001	0.0183	0.0003	0.0678	0.0605
Methionine	HMDB0000696	0.0001	0.0022	0.7564	0.0034	0.0002	0.9960	0.0004
Methylamine	HMDB0000164	0.0000	0.9290	0.0000	0.0346	0.0000	0.0067	0.0008
Oxypurinol	HMDB0000786	0.0003	0.0044	0.0217	0.0000	0.9307	0.1481	0.0589
Phenylacetate	HMDB0000209	0.0039	0.9387	0.0040	0.9971	0.0093	0.9787	0.0042
phenylalanine	HMDB0000159	0.0241	0.7825	0.9437	0.0541	0.4448	0.2496	0.0165
Pyroglutamate	HMDB0000267	0.0065	0.7031	0.0034	0.0853	0.0230	0.4369	0.3326
Pyruvate	HMDB0000243	0.0002	0.0006	0.8761	0.6530	0.0001	0.0050	0.2428
Succinate	HMDB0000254	0.0025	0.9342	0.0056	0.5134	0.0011	0.1942	0.0719
Sucrose	HMDB0000258	0.0000	0.0000	0.0000	0.0000	0.0001	0.0165	0.1200
Taurine	HMDB0000251	0.0000	0.0002	0.6773	0.0501	0.0000	0.0630	0.0042
Threonine	HMDB0000167	0.0000	0.0000	0.0000	0.7075	0.0000	0.0000	0.0000
Tyrosine	HMDB0000158	0.0065	0.4257	0.9605	0.0044	0.7246	0.0855	0.0130
Valine	HMDB0000883	0.0000	0.0009	0.0086	0.1311	0.0000	0.0928	0.0000

Supplemental Table S6. Variable Importance in Projection Scores (VIPs) Resulting from the Individual Comparison of DD groups with Respect to the IPSC Group from PLS-DA Models for the Intracellular Dataset. VIP scores >1 are presented alongside metabolite annotations and ANOVA univariate testing results. Significance level determined at * <0.05, ** <0.01, *** <0.001.

S1DD		S2DD		S3DD	
**Metabolite Annotation**	**VIP Score**	**Metabolite Annotation**	**VIP Score**	**Metabolite Annotation**	**VIP Score**
Sucrose***	1.397569	Acetylcholine***	1.555442	Acetylcholine***	1.447401
Ethanol***	1.374083	Sucrose***	1.529416	Sucrose***	1.440233
Acetate***	1.370907	Acetate***	1.504001	Creatine phosphate***	1.434273
Hydroxyvalerate***	1.369574	Ethanol***	1.498789	Homocysteine***	1.422569
Threonine***	1.337701	Alanine***	1.48808	Lactate**	1.410889
Formate***	1.312402	Hydroxyvalerate***	1.484452	Glucose-6-phosphate***	1.404792
NADP	1.278712	Glucose-6-phosphate***	1.477592	Oxypurinol***	1.402631
Deoxyadenosine	1.275282	Formate***	1.47615	Inosinic acid***	1.354926
Glucose*	1.266142	2-Hydroxybutyrate***	1.437496	Tyrosine**	1.341862
Methionine**	1.263685	Ketoleucine***	1.381863	Deoxyadenosine***	1.313858
Pyruvate***	1.241863	2-Aminobutyrate***	1.368456	Phenylalanine*	1.279026
Inosine	1.220542	Glutamate**	1.315134	Adenosine***	1.271228
Histamine	1.207087	Leucine/Alloleucine***	1.314008	Methionine*	1.244384
Glucose-6-phosphate***	1.197916	Glycine	1.288738	Valine*	1.238327
Tyrosine	1.186371	Threonine***	1.288182	Glucose	1.215356
Glycine	1.181074	2-Methylglutarate***	1.27448	Leucine/Alloleucine	1.2066
Creatine phosphate**	1.171552	Acetone***	1.259537	Lysine	1.205522
Taurine***	1.163449	Inosinic acid**	1.259403	Glycine**	1.196942
Isoleucine**	1.141893	Valine**	1.247991	Taurine	1.155978
Methylhistidine	1.126169	Indoleacetic acid*	1.239633	Indoleacetic acid	1.144108
Homocysteine***	1.105397	Pyroglutamate**	1.198366	Ethanol	1.103882
Adenosine	1.10239	Lactate***	1.18872	2-Methylglutarate	1.098045
Valine**	1.019288	Isoleucine**	1.180112	Malonate	1.084838
		Lysine**	1.13992	Ketoleucine	1.078614
		Adenosine*	1.105339	Isoleucine	1.075809
		Methylamine**	1.094791	Acetate*	1.050083
		Oxypurinol*	1.061572	Xanthine	1.033155
		Succinate**	1.00394	Creatinine	1.030859
				Methylamine	1.017562
				Histidine	1.005582

Supplemental Table S7. Variable Importance in Projection scores (VIPs) resulting from the Individual comparison of DD groups with respect to the IPSC group from PLS-DA models for the extracellular dataset. VIP scores >1 are presented alongside metabolite annotations and ANOVA univariate testing results. Significance level determined at * <0.05, ** <0.01, *** <0.001.

S1DD		S2DD		S3DD	
**Metabolite Annotation**	**VIP Score**	**Metabolite Annotation**	**VIP Score**	**Metabolite Annotation**	**VIP Score**
Formate***	1.218473	Acetate***	1.224132	Glutamate	1.2519
Glutathione	1.213458	Glutamate	1.223248	Acetate***	1.24847
Threonine***	1.212801	Creatine***	1.208352	Arginine***	1.244228
Phenylalanine***	1.211109	Arginine***	1.207134	Methanol***	1.232998
Pyruvate***	1.210975	Lactate***	1.206539	Lysine***	1.232743
Glycine***	1.209096	Choline***	1.206514	Phosphocreatine***	1.229675
Aspartate**	1.206322	Proline***	1.205668	Lactate***	1.229533
Glutamine***	1.205866	Phosphocreatine***	1.196213	Dimethylxanthine***	1.228595
Glucose*	1.203588	Glutamine***	1.194759	Creatine***	1.228266
Histidine***	1.202707	Dimethylxanthine***	1.186757	Proline***	1.228245
Glutamate	1.20054	Glucose	1.185753	Formate***	1.22189
Cysteine***	1.195824	Lysine***	1.184011	Myo inositol***	1.216357
Pyroglutamate***	1.188956	Pyruvate***	1.168333	Aspartate*	1.205373
Alanine***	1.188393	Acetylcholine***	1.153279	Choline	1.167564
Acetate***	1.184197	Valine***	1.152778	Glutamine***	1.165974
Methionine***	1.167963	2 Aminobutyrate***	1.144056	Pyroglutamate***	1.15753
Creatine***	1.155498	Methanol***	1.131734	Acetylcholine**	1.150874
Proline***	1.152625	Cysteine***	1.123402	Glycine***	1.145208
Lactate***	1.14968	Glycine***	1.121403	Alanine***	1.10391
Fructose***	1.14696	Tyrosine***	1.116766	Tyrosine***	1.075261
Threonate***	1.141046	Phenylalanine***	1.09953	Valine***	1.058319
Choline***	1.050961	Threonine***	1.087133	Homocysteine***	1.034532
Homocysteine**	1.021726	Pyroglutamate***	1.085185	Dimethylurate***	1.033392
Acetylcholine***	1.004577	Myo inositol***	1.082971	2 Aminobutyrate***	1.031598
		Alanine**	1.079592	Cysteine**	1.013555
		Leucine***	1.046988		
		Isoleucine***	1.028348		
		Ethanol**	1.028303		
		Dimethylurate***	1.018658		
		Threonate	1.01672		
		Homocysteine	1.003369		

Supplemental Table S8. Protocol for the directed differentiation (DD) of IPSCs to chondrogenic cells. IPSCs were cultured on a fibronectin matrix with DD initiated on day 1 at 90% confluence. Cells were cultured in a basal medium (DMEM:F12, 2mM L-glutamine, 1% (vol/vol) ITS, 1% (vol/vol) nonessential amino acids, 2% (vol/vol) B27, 90μM β-mercaptoethanol) temporally supplemented with growth factor combinations as shown. Differentiating cultures were expanded by passage with TrypLE™ Express at described ratios. Fibronectin = 50μg/ml; Gelatin = 0.1% (vol/vol); Fibronectin: Gelatin substrate was mixed at a ratio of 1:1. FGF2 = fibroblast growth factor-2; BMP4 = bone morphogenetic protein-4; NT4, neurotrophin-4.

Stage	Day	Matrix	Passage Ratio	Wnt3a (ng/ml)	Activin-A (ng/ml)	FGF2 (ng/ml)	Follistatin (ng/ml)	BMP4 (ng/ml)	GDF5 (ng/ml)	NT4 (ng/ml)	NMR Samples Collected
1	1	Fibronectin		25	50						
1	2	Fibronectin		25	25	20					
1	3	Fibronectin		25	10	20					
2	4	Fibronectin				20	100	40		2	Day 4
2	5	Fibronectin	1:5			20	100	40		2	
2	6	Fibronectin				20	100	40		2	
2	7	Fibronectin				20	100	40		2	
2	8	Fibronectin				20	100	40		2	
2	9	Fibronectin: Gelatin	1:2			20		20	20	2	
2	10	Fibronectin: Gelatin				20		20	20	2	
3	11	Fibronectin: Gelatin				20			40	2	Day 11
3	12	Fibronectin: Gelatin				20			40	2	
3	13	Gelatin	1:3			20			40	2	
3	14	Gelatin				20			40	2	
3	15	Gelatin				20			40	2	
3	16	Gelatin				20			40	2	
	17	Cultures Terminated									Day 17

Supplemental Table S9. Scaling factor analysis of whole spectral and methyl data for cell extract samples following normalization by total area. The table demonstrates the differences in scaling factors when applied to each sample in the dataset. Scaling factors were maintained between whole dataset and methyl data, permitting further statistical comparisons.

All Bins			Methyl Bins			Ratio of factors
**Group**	**Sample Number**	**Scaling Factor**	**Group**	**Sample Number**	**Scaling Factor**	
IPSC	1	1.372523	IPSC	1	1.421162	0.965775
	2	0.93709		2	0.995438	0.941385
	3	0.964305		3	0.985384	0.978609
	4	1.001396		4	1.079942	0.927268
	5	0.930143		5	1.023003	0.909228
DD D4	1	3.844464	DD D4	1	3.442081	1.116901
	2	3.005385		2	2.752361	1.09193
	3	3.628163		3	3.309954	1.096137
	4	2.915928		4	2.780686	1.048637
	5	1.151501		5	1.098874	1.047891
	6	2.969386		6	2.640906	1.124382
DD D11	1	0.351924	DD D11	1	0.40446	0.870108
	2	0.54798		2	0.494136	1.108966
	3	0.334604		3	0.392652	0.852165
	4	0.443504		4	0.434527	1.020661
	5	0.430913		5	0.433873	0.993176
DD D17	1	0.85096	DD D17	1	0.822874	1.034131
	2	0.831951		2	0.757282	1.0986
	3	0.818384		3	0.816293	1.002562
	4	0.82108		4	0.792287	1.036343
	5	0.624956		5	0.62982	0.992277
	6	0.878158		6	0.811288	1.082424
EB D4	1	0.142086	EB D4	1	0.282962	0.502139
	2	0.16573		2	0.31541	0.525442
	3	0.178749		3	0.353217	0.506061
	4	0.160837		4	0.295878	0.543593
	5	0.131821		5	0.270455	0.487406
	6	0.201494		6	0.316388	0.636856
EB D17	1	0.141895	EB D17	1	0.288409	0.491993
	2	0.134606		2	0.289319	0.465252
	3	0.088082		3	0.268679	0.327834

Supplemental Table S10. Summary Statistics for all PLS-DA Models Employed. 10-fold CV performed per PLS-DA to calculate accuracy, R2, Q2, and train:test splits employed to determine AUROC as per Szymanska et al 2012 [78] and 2000 permutations of each PLS-DA performed via metaboanalyst (Xia et al 2009 [79]).

	**Accuracy**	**R2**	**Q2**	**CV-ANOVA**	**ROC**	**Permutation Results p-value (permutations)**	**Number of Components**
**Figure 1 c**	0.94118	0.96012	0.84209	***	1	< 5e-04 (0/2000)	2
**Supplemental 1 c (I v D)**	1	0.98326	0.93863	***	1	0.015 (30/2000)	2
**Supplemental 1 c (I v S)**	0.90909	0.96613	0.81582	***	1	0.0215 (43/2000)	2
**Figure 1 d**	1	0.99837	0.9975	***	1	< 5e-04 (0/2000)	2
**Supplemental 1 d (I v D)**	1	0.99827	0.99454	***	1	0.0015 (3/2000)	2
**Supplemental 1 d (I v S)**	1	0.99966	0.99913	***	1	0.0265 (53/2000)	2
**Figure 3 c**	0.95455	0.96835	0.91724	***	1,1,1,0.75 (DD3 v others)	< 5e-04 (0/2000)	3
**Supplemental 3 c (I v D1)**	1	0.98133	0.93997	***	1	0.0485 (97/2000)	2
**Supplemental 3 c (I v D2)**	1	0.97815	0.93086	***	1	0.0385 (77/2000)	2
**Supplemental 3 c (I v D3)**	0.90909	0.99201	0.87501	***	1	0.22 (440/2000)	2
**Figure 3 d**	1	0.98284	0.96574	***	1,1,1,0.75 (DD3 v others)	< 5e-04 (0/2000)	2
**Supplemental 3 d (I v D1)**	1	0.99888	0.98962	***	1	0.0065 (13/2000)	2
**Supplemental 3 d (I v D2)**	1	0.99687	0.992	***	1	0.0185 (37/2000)	2

**Supplemental 3 d (I v D3)**	1	0.99702	0.99069	***	1	0.0515 (103/2000)	2

*** p-val <1e-6 from 10-fold CV-ANOVA from a linear regression of same group/metabolite comparison, using caret [Max Kuhn (2022). caret: Classification and Regression Training. R package version 6.0-92].

References:

E. Szymanska E. Saccenti A. K. Smilde J. A. Westerhuis Double-check: validation of diagnostic statistics for PLS-DA models in metabolomics studies Metabolomics (2012) 8:S3–S16 DOI 10.1007/s11306-011-0330-3

Xia, J., Psychogios, N., Young, N. and Wishart, D.S. (2009) MetaboAnalyst: a web server for metabolomic data analysis and interpretation. Nucl. Acids Res. 37, W652-660.
